# Selected traditional Chinese medicine interventions for post-stroke cerebral edema: a review integrating clinical evidence and mechanistic insights

**DOI:** 10.3389/fphar.2025.1709821

**Published:** 2025-12-10

**Authors:** Junyue Huang, Yujia Jin, Yuping Chen, Mengsi Wang, Jian Wu

**Affiliations:** 1 The Third School of Clinical Medicine, Zhejiang Chinese Medical University, Hangzhou, Zhejiang, China; 2 Department of Neurology, The Second Affiliated Hospital, Zhejiang University School of Medicine, Hangzhou, Zhejiang, China; 3 Department of Neurology, The Third Affiliated Hospital of Zhejiang Chinese Medical University, Hangzhou, Zhejiang, China; 4 Department of Neurology, Tiantai People’s Hospital of Zhejiang Province (Tiantai Branch of Zhejiang Provincial People’s Hospital), Hangzhou Medical College, Taizhou, Zhejiang, China

**Keywords:** stroke, cerebral edema, traditional Chinese medicine, safety, botanical drug

## Abstract

**Background:**

Stroke is a leading cause of global death and disability. Post-stroke cerebral edema significantly worsens neurological outcomes. While conventional therapies face safety limitations, selected traditional Chinese medicine (TCM) interventions offer a potential alternative.

**Aim of the study:**

This narrative review aims to comprehensively evaluate the efficacy and safety of TCM interventions for post-stroke cerebral edema and elucidate their potential mechanisms based on experimental evidence.

**Materials and methods:**

A systematic literature search was conducted in PubMed, Web of Science, and other databases using keywords related to stroke, cerebral edema, and TCM interventions. Studies were screened according to predefined inclusion criteria to ensure methodological rigor.

**Results:**

Clinical and preclinical studies indicate that TCM interventions can reduce cerebral edema volume, improve neurological outcomes, and exhibit good safety. These effects may be associated with modulation of ion homeostasis and aquaporins, neuroinflammatory inhibition, blood-brain barrier protection, oxidative stress reduction, and apoptosis suppression.

**Conclusion:**

Selected TCM interventions show promise for post-stroke cerebral edema. Their clinical experience and mechanistic insights provide a valuable foundation for future research and drug development.

## Graphical Abstract

**Figure:**
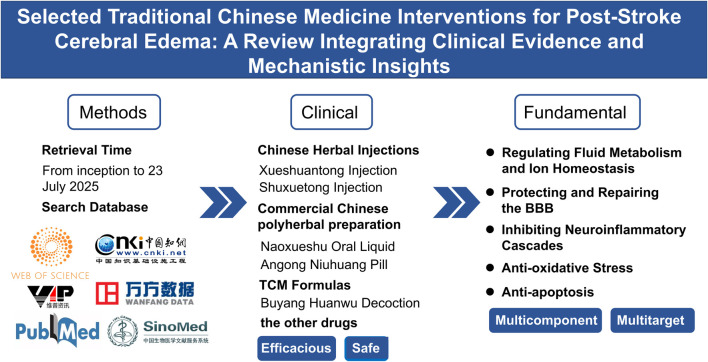

## 1 Introduction

Stroke, a primary global cause of death and severe long-term disability, is frequently complicated by cerebral edema—a potentially fatal condition affecting 10%–78% of ischemic and hemorrhagic stroke patients (Jiang et al., 2022). Each 1 mL increase in edema volume elevates the risk of adverse outcomes by 8.0%, exacerbating neurological deficits and mortality (Nawabi et al., 2019). BBB disruption is the most common etiology. Mannitol remains the first-line osmotic diuretic clinically, but carries significant limitations: high doses risk osmotic nephropathy, and rapid renal excretion can cause rebound intracranial hypertension (Cook et al., 2020; Papagianni et al., 2018). Decompressive craniectomy effectively reduces mortality but often leaves severe disability (Cooper et al., 2011). Consequently, clinical management of post-stroke cerebral edema remains challenging, driving increasing patient interest in TCM as an alternative or adjunctive therapy seeking improved efficacy and reduced adverse effects.

In China, TCM has been used to treat stroke for over 2000 years, demonstrating notable efficacy and safety (Zhao et al., 2024). Recent research increasingly reveals its potential for post-stroke edema management. Numerous botanical drugs, formulas, and metabolites have been shown to reduce brain swelling, lower intracranial pressure, and improve neurological function in preclinical models and early clinical trials (Li X. et al., 2020; Widmann et al., 2018). Compared to mannitol or surgery, TCM therapies generally present lower risks of adverse reactions, making them particularly attractive for patients with comorbidities such as cardiac or renal insufficiency or diabetes (Lin et al., 2022). However, the clinical evidence and mechanisms underlying TCM require further elucidation. This article is presented as a narrative review that synthesizes current advances in the clinical efficacy and pharmacological mechanisms of TCM for post-stroke cerebral edema, aiming to guide clinical application and accelerate drug development.

## 2 Methods

In this review, we conducted a comprehensive literature search across six databases—Web of Science, PubMed, China National Knowledge Infrastructure (CNKI), VIP, Wanfang, and SinoMed—from inception to 23 July 2025 to evaluate the efficacy and safety of TCM for post-stroke cerebral edema. Key search terms included: “stroke,” “cerebral ischemia,” “intracerebral hemorrhage,” “edema,” “traditional Chinese medicine,” “herbal,” “TCM,” “formula,” “Clinical” and “patient.” Detailed search strategies for each database are listed in Supplementary.

For clinical studies, the exclusion criteria were as follows: (a) case reports, reviews, commentaries, and animal studies; (b) studies involving non-ischemic or non-hemorrhagic stroke patients; (c) complementary and alternative therapies other than TCM, including acupuncture, moxibustion, cupping, massage, qigong, tai chi, yoga, and music therapy; and (d) studies without reported clinical outcomes.

For preclinical studies, the exclusion criteria were as follows: (a) case reports, reviews, commentaries, and clinical studies; (b) studies using cell or animal models unrelated to stroke; (c) studies involving complementary and alternative therapies other than TCM, such as acupuncture, cupping, massage, and music therapy; and (d) studies lacking experimental outcome measures related to cerebral edema.

## 3 Clinical evidence for selected TCM interventions in post-stroke cerebral edema

### 3.1 Chinese botanical drug injections

Chinese herbal injections are modern TCM preparations made from effective substances extracted from Chinese medicines (single botanical drugs or compound formulas), formulated as sterile solutions for intravenous or intramuscular injection to directly enter the systemic circulation.

Xueshuantong Injection is a standardized TCM product extracted from *Panax notoginseng* (Burkill) F.H.Chen, with total saponins of *P. notoginseng* as its main metabolites. *Panax notoginseng* is widely used for promoting blood circulation and resolving stasis in the treatment of post-stroke cerebral edema. Pharmacological studies have shown that *P. notoginseng* can improve cerebral blood flow, protect vascular endothelial function, and scavenge free radicals (Pan et al., 2022). In clinical cases, Xueshuantong Injection has been shown to reduce the volume of cerebral edema and hematoma, improve hemodynamics, and decrease infarct volume (He et al., 2002). A systematic review of 21 clinical studies involving 1759 patients demonstrated that Xueshuantong exerts a protective effect on brain tissue during acute intracerebral hemorrhage and is more effective than the control group in reducing cerebral edema (Yu et al., 2011). A 160-patient clinical trial showed that compared with conventional treatment alone, Xueshuantong Injection combined with conventional treatment reduced edema volume and infarct size, enhanced anti-inflammatory responses, promoted neurological recovery, lowered disability rates, and improved quality of life and prognosis in patients with post-hemorrhagic stroke edema (Wang, 2018). Another clinical trial involving 115 patients reported that Xueshuantong Injection had a relatively safe, with an adverse reaction rate of 5.26% in the observation group and 3.45% in the control group. The main adverse reactions include gastrointestinal discomfort, skin rashes, fatigue, and other allergic reactions (Shang et al., 2022). The molecular biological mechanism of Xueshuantong Injection involves regulating the HIF1-α/VEGFA/VEGFR2 signaling pathway to promote vascular remodeling in the ischemic area after stroke, thereby reducing BBB leakage (Fang et al., 2023; Hatakeyama et al., 2020). Transcriptomic and proteomic analyses further revealed that Xueshuantong Injection significantly regulates the expression of related proteins and genes (VEGFA, VEGFR2, AKT, p-AKT, HIF-1α, GAPDH, EGFR, IL-6, HSP90AA1, NFKB1, PTEN) in middle cerebral artery occlusion (MCAO) model rats, suggesting that the HIF1-α/VEGFA/VEGFR2 signaling pathway is a potential key pathway for alleviating post-ischemic cerebral edema (Xiao et al., 2024).

Shuxuetong Injection is a sterile aqueous solution extracted from animal-derived medicinal materials *Hirudo nipponia* Whitman (leech) and *Pheretima aspergillum* (E. Perrier) using modern biotechnology. Its main metabolites include hirudin-like polypeptides, earthworm fibrinolytic enzymes, and collagen hydrolysates (Sun et al., 2022). An 85-patient trial in intracerebral hemorrhage (ICH) patients showed that Shuxuetong Injection improved cerebral edema severity, reduced hematoma volume, enhanced NIHSS scores, and promoted neurological recovery in post-hemorrhagic cerebral edema, with no significant adverse effects reported (Yao, 2012). In addition, another clinical trial involving 120 patients with intracerebral hemorrhage showed that compared with conventional treatment, Shuxuetong Injection combined with conventional treatment was more effective in reducing the volume of cerebral edema and hematoma (Liang and Yang, 2012). Mechanistic insights from preclinical models propose that Shuxuetong may alleviate cerebral edema by inhibiting pyroptosis, a pro-inflammatory form of cell death, potentially *via* the CD44/NLRP3/GSDMD signaling pathway (Shang et al., 2025).

### 3.2 Commercial Chinese polyherbal preparation (CCPP)

Commercial Chinese polyherbal preparation are standardized TCM preparations produced in batches with fixed formulas and processes, using Chinese medicinal materials as raw materials, and having specific dosage forms and efficacy.

Naoxueshu Oral Liquid, a clinically utilized Chinese CCPP for qi-supplementing, blood-activating, stasis-resolving, and fluid-promoting effects, contains components including *Astragalus mongholicus* Bunge, *H. nipponia* Whitman, *Acorus tatarinowii* Schott, *Achyranthes bidentata* Blume, *Paeonia suffruticosa* Andr., *Rheum officinale* Baill., and *Ligusticum chuanxiong* Hort (Li P. et al., 2024). Modern studies on its active substances have identified over 100 distinct metabolites in the formula, which exert synergistic multi-target effects to facilitate edema absorption and neurological recovery after stroke (Li R. et al., 2024). A network meta-analysis of 19 studies (n = 2,335 patients) demonstrated that Naoxueshu combined with Western medicines (nimodipine, nifedipine, edaravone) significantly reduced cerebral edema volume and hematoma size, improved NIHSS scores, and exerted anti-inflammatory effects compared to Western medicines alone (Mei et al., 2022). A 220-patient trial showed that Naoxueshu plus conventional treatment Moderately decreased edema and hematoma volume while improving neurological function scores *versus* conventional treatment alone (Song et al., 2022). In addition, A 120-patient study in post-stroke rehabilitation reported that 7-day Naoxueshu treatment reduced cerebral hematoma and edema volume without affecting fibrinogen levels or causing adverse effects (Song et al., 2021). Experimental research suggests potential mechanisms for these benefits. Studies in rat models of intracerebral hemorrhage indicate that Naoxueshu may reduce BBB permeability and alleviate edema, potentially through modulating astrocyte function, promoting the expression of the tight junction protein ZO-1, and inhibiting AQP4 protein (Wang et al., 2019).

Angong Niuhuang Pill is a commercially available Chinese CCPP. This pill is composed of 11 medicinal components: *Bos taurus domesticus* Gmelin, *Moschus moschiferus* Linnaeus, *Bubalus bubalis* Linnaeus (horn, concentrated powder), *Pinctada martensii* (Dunker), *Cinnabaris*, *Realgar*, *Coptis chinensis* Franch., *Scutellaria baicalensis* Georgi, *Gardenia jasminoides* Ellis, *Curcuma aromatica* Salisb., and *Dryobalanops aromatica* Gaertn. f (Bai et al., 2024). An 80-patient trial in acute cerebral infarction (ACI) demonstrated that Angong Niuhuang Pill combined with conventional treatment significantly reduced edema volume and improved NIHSS and GCS scores compared to conventional treatment alone (Feng and Yang, 2015). In addition, A larger 122-patient trial in ACI reported that the pill restored midline shift, Significantly decreased edema volume, modulated vascular tone, and enhanced neurological function and quality of life (Zhang and Chen, 2016). Notably, realgar and cinnabar—key components—contributed to neuroprotective effects without inducing hepatotoxicity or nephrotoxicity in transient ischemic brain injury models (Tsoi et al., 2019a).

Systems biology approaches have shown that Angong Niuhuang Pill can effectively improve cerebrovascular edema in mice, which may be associated with the inhibition of VE-cadherin expression and the upregulation of CAV-1 phosphorylation and AQP4 expression in mouse brain tissue (Liu et al., 2024). Another study suggested a potential mechanism involving the prevention of MMP-9 activation *via* scavenging peroxynitrite, thereby protecting microvascular integrity (Chen H. et al., 2022).

### 3.3 Chinese medicinal formulae

Chinese Medicinal Formulae represent systematic combinations of multiple herbs—often guided by principles such as “monarch, minister, assistant, and guide”—with the goal of producing synergistic therapeutic effects.

Buyang Huanwu Decoction, derived from *Yilin Gaicuo* by Wang Qingren in the Qing Dynasty, is a classic formula for treating post-stroke sequelae, composed of seven medicinal components: *Astragalus membranaceus* (Fisch.) Bunge, *Angelica sinensis* (Oliv.) Diels, *Paeonia lactiflora* Pall., *L. chuanxiong* Hort., *P. aspergillum* (E. Perrier), *Prunus persica* (L.) Batsch, and *Carthamus tinctorius* L. Its standardized extract contains core metabolites such as astragaloside IV, ferulic acid, paeoniflorin, and hydroxysafflor yellow A (Pan et al., 2025). A 104-patient trial post-intracerebral hemorrhage surgery showed that Buyang Huanwu Decoction combined with ultra-early urokinase significantly reduced cerebral edema probability, increased overall response rates, and demonstrated a favorable safety profile compared to urokinase alone (Hou et al., 2022). Another 52-patient study in ACI reported that Buyang Huanwu Decoction can reduce the content of β2-MG in CSF of patients with acute cerebral infarction, alleviate cerebral edema, and has reliable clinical efficacy (Jia and Liu, 2007; Xiao et al., 2021). Separately, research suggests it may prevent cerebral edema after ischemia by inhibiting the HIF-1α/VEGF pathway and stabilizing β-ENaC ion channels in the brain.

Preclinical studies have proposed multiple potential mechanisms. In rat models, its effects have been associated with the inhibition of the NIK-mediated non-canonical NF-κB pathway to reduce inflammatory responses after intracerebral hemorrhage (Xiao et al., 2021). Separately, research suggests it may prevent cerebral edema after ischemia by inhibiting the HIF-1α/VEGF pathway and stabilizing β-ENaC ion channels in the brain (Chen et al., 2019).

Clinical reports on Chinese Botanical Drug Injections, commercial Chinese polyherbal preparation and TCM Formulas are summarized in Table 1.

**TABLE 1:** Clinical reports related to the treatment of post-stroke cerebral edema with selected TCM interventions.

Disease	Prescription	Sample size	Treatment duration	Experimental group	Control group	Edema volume (experimental/control group)	Outcome indicators	Adverse reactions	Author(s) and year
Cerebral hemorrhage	Xueshuantong injection	160	14 days	Routine treatment + Xueshuantong Injection 4 mL ivgtt qd	Routine treatment	75.86%	hs-CRP ↓, MMP-9 ↓, MBP ↓, NSE ↓, S100B protein ↓	N	Wang (2018)
	Shuxuetong injection	120	14 days	Routine treatment + Shuxuetong Injection 4 mL ivgtt qd	Routine treatment	48.27%	hematoma volume ↓, fibrinogen ↓	N	Liang and Yang (2012)
	Danshen injection	120	14 days	Routine treatment + Nimodipine + Danshen Injection 16 mL ivgtt tid	Routine treatment + Nimodipine	59.54%	ESS ↑	N	Zeng (2015)
	Erigeron breviscapus injection	100	14 days	Routine treatment + Edaravone + Erigeron Breviscapus Injection 40 mL ivgtt qd	Routine treatment + Edaravone	78.47%	cerebral hematoma volume ↓, NIHSS ↑, IL-18 ↓, HSP47 ↓, CD163 ↓, S100β ↓	N	Wei et al. (2024)
	Naoxueshu oral liquid	120	7 days	Routine treatment + Naoxueshu Oral Liquid 10 mL po tid	Routine treatment	86.2%	hematoma volume ↓, NIHSS ↓	N	Song et al. (2022)
	Jianshen lishui granule	100	14 days	Routine treatment + Furosemide + Jianshen Lishui Granule 91g po tid	Routine treatment + Furosemide	49.35%	hematoma volume ↓, NIHSS ↓, hs-CRP ↓, S-100β protein ↓, IL-1β ↓	Y	Tan et al. (2021)
	Longhu xingnao granule	25	14 days	Torasemide + Mannitol + Longhu Xingnao Granule 10g po bid	Torasemide + Mannitol	70.39%	hematoma volume ↓, NIHSS ↓, ADL ↑	N	Yu et al. (2018)
	Huoxue tongli decoction	170	21 days	Routine treatment + Huoxue Tongli Decoction 100 mL po bid	Routine treatment	61.90%	hematoma absorption rate ↑	N	Zhao et al. (2010)
	Huoxue ditan decoction	106	14 days	Routine treatment + Hyperbaric oxygen + Huoxue Ditan Decoction 100 mL po bid	Routine treatment + Hyperbaric oxygen	76.42%	NIHSS ↑, ET ↓, NO ↓, IL-1 ↓	N	Gao et al. (2020)
	Changpu yujin decoction	130	30 days	Routine treatment + Changpu Yujin Decoction 50 mL Nasogastric Gavage q4h	Routine treatment	83.69%	hs-CRP ↓, NSE ↓, FIB ↓	N	Fu et al. (2019)
	Sanyu tongluo decoction	120	20 days	Routine treatment + Sanyu Tongluo Decoction 100 mL po qd	Routine treatment	65.45%	cerebral hematoma ↓, NIHSS ↓, BI ↑, IL-6 ↓, IL-4 ↓, BDNF ↓, VEGF ↓	N	Yu et al. (2023)
	Liangxue sanyu decoction	72	14 days	Nimodipine + Liangxue Sanyu Decoction 95g po bid	Routine treatment + Nimodipine	81.24%	NIHSS ↓, BI ↑	N	Zhong et al. (2019)
	Lingjiao gouteng decoction	102	3 months	Routine treatment + Lingjiao Gouteng Decoction 90g po bid	Routine treatment	73.83%	hematoma volume ↓, NIHSS ↓, PRO ↓, Barthel ↑, modified Rankin ↓	N	Jiao and Xu (2015)
Cerebral ischemia	Angong niuhuang pill	122	14 days	Routine treatment + Angong Niuhuang Pill 3g po qd	Routine treatment	60.22%	recovery of midline shift ↑, GCS ↑, NIHSS ↓, ADMA ↓, NO ↑	N	Zhang and Chen (2016)
	Buyang huanwu decoction	52	14 days	Routine treatment + Buyang Huanwu Decoction 100 mL po bid	Routine treatment	66.88%	β2-MG ↓	N	Jia and Liu (2007)
	Xiaoxuming decoction	90	14 days	Routine treatment + Xiaoxuming Decoction 100 mL po bid	Routine treatment	71.25%	S-100β ↓, NSE ↓, MBP ↓, NIHSS ↓	N	Zhao et al. (2023)
	Xinglou chengqi decoction	50	7 days	Routine treatment + Xinglou Chengqi Decoction 62g po bid	Routine treatment	73.33%	cerebral hematoma volume ↓, NIHSS ↑	N	Fan (2019)

^a^ **Routine treatment:** Includes antiplatelet therapy, antihypertensive therapy, lipid regulation, improvement of circulation and neuroprotection. The specific components of routine treatment may vary slightly among different studies.

### 3.4 Adverse reactions

In recent years, With the expanding clinical application of TCM, reports of adverse reactions have correspondingly increased. The most common reactions involve the skin, digestive, and respiratory systems, manifesting as pruritus, rashes, flatulence, fatigue, vomiting, headache, and dizziness. Primary management strategies include drug discontinuation and symptomatic treatment (Lai et al., 2013). A meta-analysis of 40 RCTs (n = 3,868) showed no significant difference in adverse reaction rates between Xueshuantong plus conventional therapy (12.5%) and conventional therapy alone (11.4%) (Lyu et al., 2020). Another systematic review of Naoxueshu Injection for ICH (n = 1,602) reported significantly reduced cerebral edema in the treatment group *versus* controls, with no abnormalities in safety parameters (blood/coagulation profiles, liver/kidney function) and no mortality difference (Wang et al., 2025). Animal toxicology studies demonstrated that rats administered Angong Niuhuang Pill at human-equivalent doses exhibited no mercury accumulation in blood, liver, or kidneys within 7–14 days; although arsenic levels increased in the liver and blood (not kidneys), short-term use (≤7 days) remained safe (Tsoi et al., 2019b). However, most of the included clinical studies did not use standardized reporting protocols for adverse events, and some studies did not report adverse reactions at all, which may limit the comparability and interpretation of safety data across studies. In summary, Selected TCM interventions exhibits an acceptable safety profile in clinical practice. Future efforts should prioritize standardized adverse event reporting, rigorous safety evaluations, and long-term follow-up in large-scale trials to further strengthen the reliability of safety data.

## 4 Pharmacological mechanisms of selected TCM interventions in post-stroke cerebral edema

The pathological mechanisms of cerebral edema after stroke differ essentially between intracerebral hemorrhage and cerebral ischemia. Intracerebral hemorrhage drives progressive vasogenic edema through thrombin-mediated endothelial injury combined with iron-catalyzed Fenton reactions from hemoglobin degradation, which disrupt BBB integrity. Cerebral ischemia initially induces cytotoxic edema due to sodium-potassium pump dysfunction caused by energy failure, and then transforms into vasogenic edema during reperfusion as reactive oxygen species (ROS) bursts activate matrix metalloproteinases (MMPs) to degrade tight junction proteins. The treatment of cytotoxic edema primarily involves regulating fluid metabolism and ion homeostasis, while the key to treating vasogenic edema lies in protecting and repairing the BBB (Wang et al., 2024a). The treatment of both types of edema requires inhibiting the neuroinflammatory cascade to block the transformation of cytotoxic edema to vasogenic edema, ultimately achieving edema resolution (Zheng et al., 2023).

**FIGURE 1:**
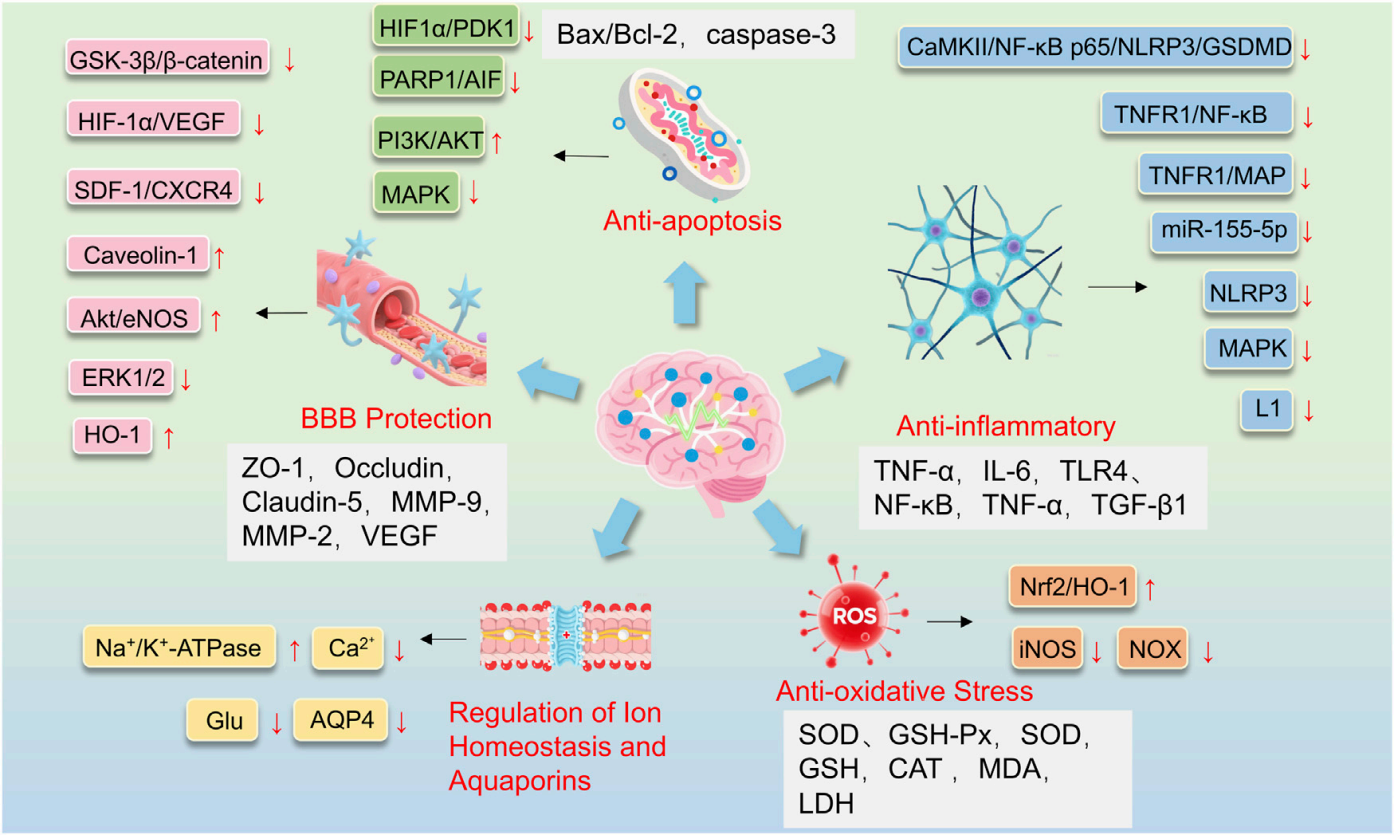
Pharmacological mechanisms of selected traditional Chinese medicine interventions for post-stroke cerebral edema.

Preclinical evidence suggests that selected TCM interventions may ameliorate cerebral edema by targeting interconnected pathological processes. Evidence for their potential mechanisms of action comes primarily from animal and cell-based models, and points to several key processes: the regulation of ion homeostasis and aquaporins, inhibition of neuroinflammatory cascades, protection and repair of the BBB, and the mitigation of oxidative stress and apoptosis. These potential mechanisms are illustrated in Figure 1. Basic research on TCM monomers and Chinese Herbal Formulas are presented in Tables 2, 3, respectively. It is important to note that this evidence currently constitutes a framework of hypotheses that must be rigorously tested. The critical next steps for the field will involve establishing causal validation of these mechanisms in humans and identifying the principal active constituents within the complex formulas. Building on this foundation, the following sections will elaborate on each of these mechanisms.

**TABLE 2:** Statistics of basic experimental studies on the treatment of post-stroke cerebral edema with Chinese herbal metabolite.

Effect	Source	Active component	Structure	Animal/cells	Treatment	Targets	References
Regulating fluid metabolism and ion homeostasis	*Rheum palmatum* L.	Emodin	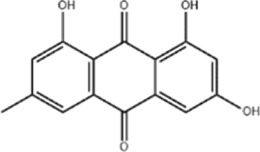	SD rat Primary rat cortical astrocytes	*In vivo*: MCAO rats received intravenous emodin (15 mg/kg) at 8 and 12 h post-reperfusion, with outcomes measured at 24 h. *In vitro*: Cells were pre-treated with liposomal emodin for 24 h prior to OGD/R.	Brain water content↓, AQP4 ↓, ZO-1↑, Claudin 1↑, TNF-α↓, IL-1β↓, IL-6↓	Chen et al. (2024)
	*Cornus officinalis* Sieb. et Zucc	Cornel Iridoid glycoside	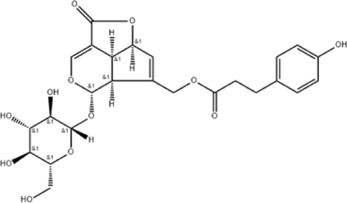	SD rat	*In vivo*: MCAO/R rats were pre-treated with CIG (15 and 30 mg/kg, i.g.) for 3 days and once at 2 h before ischemia, with outcomes measured at 24 h post-reperfusion.	Brain water content↓, AQP4 ↓, pyknotic nucleus ↓, vacuole degeneration↓	Wang et al. (2020)
	*Dioscorea zingiberensis* C.H.Wright	Total steroid saponins	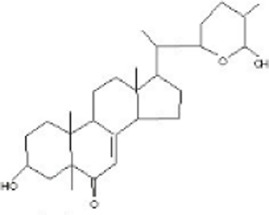	SD rat	*In vivo*: MCAO/R rats were pre-treated with TSSN (30, 10, and 3 mg/kg, i.g.) or Nimodipine (20 mg/kg, i.g.) once daily for 7 days before ischemia, with outcomes measured at 24 h post-reperfusion.	Brain water content↓, AQP-4↓, neurological deficit scores↑, cerebral infarct size↓, CAT↑, SOD↑, NO↓, MDA↓, iNOS↓, NF-κB↓, ERK 1/2↓	Zhang et al. (2014)
	*Gastrodia elata* Blume	Phenolic glucoside gastrodin	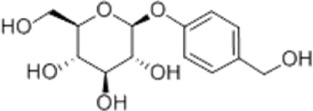	SD rat, rat hippocampal neurons	*In vivo*: MCAO rats received intraperitoneal gastrodin (100 and 50 mg/kg) at the onset of ischemia, with outcomes measured at 24 h post-reperfusion. *In vitro*: Cultured hippocampal neurons were treated with gastrodin (30 and 15 μg/mL) during OGD/glutamate exposure and the subsequent 24-h period.	Brain water content↓, cerebral infarct size↓, Ca2+↓, NO↓, glutamic acid↓	Zeng et al. (2006).
Protecting and repairing the BBB	*Citrus paradisi* Macfadyen *Citrus aurantium* L.	Naringenin	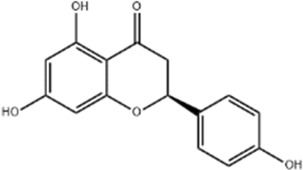	C57BL/6J mice	*In vivo*: MCAO mice received intraperitoneal NAR (10 mg/kg) once daily for 6 days immediately after reperfusion, with outcomes measured at 7 days.	Cerebral infarct size↓, ZO-1↑, Occludin↑, Claudin-5↑, IL-1β↓, IL-6↓, TNF-α↓, β-catenin↑, p-GSK-3β (ser-9)↑	Yang et al. (2024)
	*Curcuma longa* L.	Curcumin	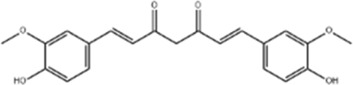	RBMEC	*In vitro*: RBMECs were treated with curcumin (20 μM) during OGD for 24 h.	Brain water content↓, Occludin↑, ZO-1↑, HO-1↓	Wang et al. (2013)
	*Scutellaria baicalensis* Georgi	Baicalin	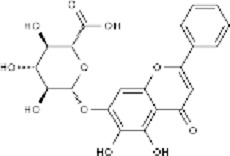	BMVECs	*In vitro*: BMVECs were treated with baicalin (5, 10, 20 μg/mL) during OGD for 24 h.	brain water content↓, Oclaudin-5↑, ZO-1↑, PKC↓	Zhu et al. (2012)
	*Crocus sativus* L.	Crocin	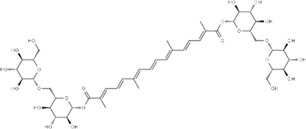	C57BL/6J mice	*In vivo*: BCCAO mice received crocin (20, 10, 5 mg/kg) intragastrically once daily for 21 days before I/R, with outcomes measured at 24 h post-reperfusion.	Brain water content↓, NO↓, MDA↓, SOD↑, GSH-px↑, MMP-9↓, ERK1/2 phosphorylation↓	Zheng et al. (2007)
	*Coffea arabica* L. *Cichorium intybus* L.	Dihydrocaffeic acid	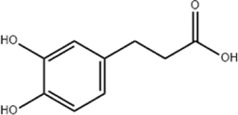	SD rat	*In vivo*: tMCAo rats received intraperitoneal DHCA (3, 10, 30 mg/kg) at 0 and 2 h after onset of ischemia, with outcomes measured at 24 h after ischemia.	Brain water content↓, cerebral infarct size↓, EB extravasation↓, MMP-2↓, MMP-9↓	Lee et al. (2015)
	*Panax ginseng* C. A. Mey.	Ginsenoside Rd	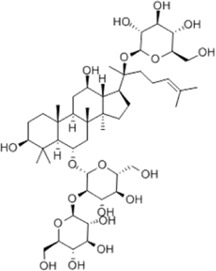	SD rat	*In vivo*: MCAO rats received intraperitoneal Ginsenoside Rd (30 mg/kg) 1 h before ischemia, with outcomes measured at 24 h after MCAO.	Brain water content↓, NF-κB↓, MMP-9↓	Zhang et al. (2020)
	*Curcuma longa* L.	Tetrahydrocurcumin	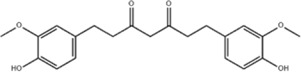	CBS heterozygous knockout mice	*In vivo*: MCAO mice received intraperitoneal THC (25 mg/kg in 0.1% DMSO) daily for 3 days starting at 30 min post-ischemia, with outcomes measured at 72 h post-reperfusion.	Brain water content↓, Hcy ↓, MMP-9 ↓, LC3-II↓, DRAM↓	Tyagi et al. (2012).
Inhibiting neuroinflammatory cascades	*Rehmannia glutinosa* Libosch, *Ligustrum lucidum* Ait., *Plantago lanceolata* L., *Verbena officinalis* L.	Verbascoside	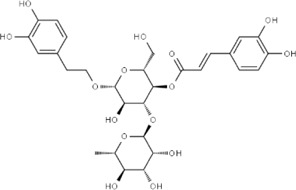	C57BL/6J mice	*In vivo*: ICH mice received intraperitoneal VB (15 or 30 mg/kg) 15 min post-ICH, with outcomes measured at 24, 48, and 72 h.	Brain water content↓, TLR4↓, NF-κB↓, TNF-α↓, IL-1β↓, IL-6↓	Lai et al. (2019)
	*Ginkgo biloba* L.	Ginkgolide C	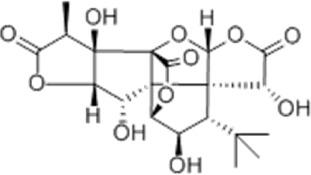	SD rat	*In vivo*: MCAO/R rats received intraperitoneal GC (8, 16, or 32 mg/kg) daily for 3 days starting at reperfusion, with outcomes measured at 72 h post-reperfusion.	Brain water content↓, cerebral infarct size↓, MPO↑, ICAM-1↓, VCAM-1↓, iNOS ↓, CD40↓, TNF-α↓, IL-1β↓, IL-6↓	Li et al. (2023)
	*Vitis vinifera* L., *Pinus pinaster* Aiton, *Nelumbo nucifera* Gaertn	Procyanidins	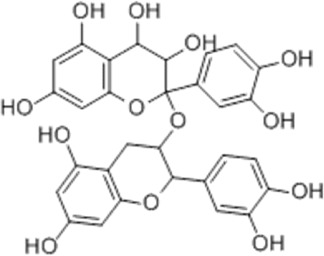	SD rat BV-2cells	*In vivo*: MCAO/R rats received intragastric procyanidins (20, 40, or 80 mg/kg) 1 h before ischemia, with outcomes measured at 24 h post-reperfusion. *In vitro*: BV2 cells were pre-treated with procyanidins (10 μM) for 2 h prior to OGD/R.	Brain water content↓, cerebral infarct size↓, TLR4↓,p38↓, NF-κB↓, NLRP3↓	Yang et al. (2020)
	*Ginkgo biloba* L.	Ginkgo diterpene lactones	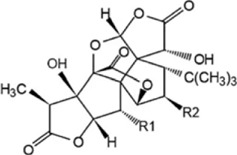	SD rat Cortical astrocytes	*In vivo*: MCAO/R rats received intravenous GDL (1.25 mg/kg) at 1 h after reperfusion, twice daily for 3 days, with outcomes measured at 72 h after MCAO. *In vitro*: Astrocytes were pre-treated with GDL (12.5, 25, 50 μg/mL) for 24 h prior to OGD/R.	Brain water content↓, cerebral infarct size↓, IL-1β↓, TNF-α↓, TLR4 ↓, NF-κB, LDH↓	Li Z. et al. (2020)
	*Paeonia suffruticosa* Andr., *Cynanchum paniculatum* (Bunge) Kitagawa	paeonol	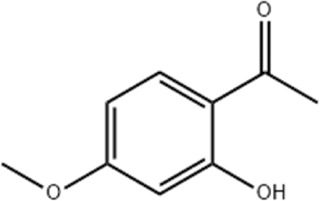	SD rat	*In vivo*: MCAO rats received intraperitoneal paeonol (25 mg/kg) at the onset of ischemia and then once daily, with outcomes measured at 72 h and 28 days after reperfusion.	Brain water content↓, cerebral infarct size↓, astrocytes↓, Iba-1-positive microglia↓	Zhao et al. (2018)
	*Ferula sinkiangensis* K. M. Shen	Kellerin	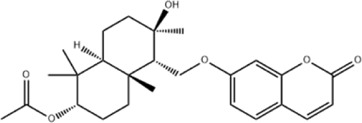	SD rat BV2 and SH-SY5Y cells	*In vivo*: MCAO rats received intragastric kellerin (3.5, 7.0, 14.0 mg/kg) at the onset of reperfusion, with outcomes measured at 24 h after reperfusion. *In vitro*: Cells were pre-treated with kellerin (1, 5, 10 µM) for 2 h prior to LPS stimulation.	Brain water content↓, cerebral infarct size↓, NO↓, iNOS↓, NF-κB↓, ROS↓, NADPH↓	Mi et al. (2021)
	*Artemisia annua* L.	Artemisinin	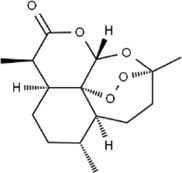	C57BL/6J mice	*In vivo*: ICH mice received intraperitoneal artemisinin (5 mg/kg) once daily, with outcomes measured at 24 h and 72 h after ICH.	Brain water content↓, L1↓, ROS↓, GSH↓, SOD↓, IL-1β↓, IL-6↓, TNF-α↓	Wang et al. (2022a)
Anti-oxidative stress	Olea europaea L., Ligustrum lucidum Ait.	oleanolic acid	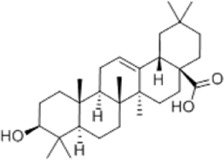	Primary cultured cortical neurons	*In vitro*: Cultured rat cortical neurons were pre-treated with Aralia cordata extract (5-20 μg/mL) or Oleanolic Acid (0.5-5 µM) for 15 min prior to H2O2 exposure.	Brain water content↓, cerebral infarct size↓, Ca2+↓, ROS↓	Cho et al. (2009)
	Rehmannia glutinosa (Gaertn.) DC., Picrorhiza scrophulariiflora Pennell, Scrophularia ningpoensis Hemsl.	Catalpol	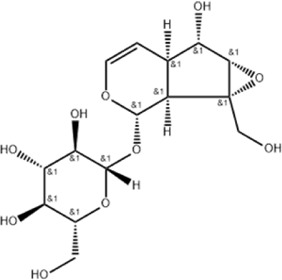	SD rat	*In vivo*: MCAO rats received Catalpol (2.5, 5, 10 mg/kg, intranasal) for 3 days after modeling, with outcomes measured at 72 h post-reperfusion.	Brain water content↓, cerebral infarct size↓, SOD↓, MDA↓, Bcl-2↓, Bax↑, Nrf2↑, HO-1↑	Wang et al. (2022b)
	*Astragalus membranaceus* (Fisch.) Bge. var. mongholicus (Bge.) Hsiao	Astragaloside IV	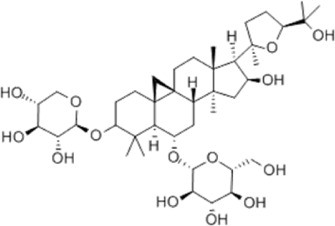	SD rat	*In vivo*: MCAO rats received Astragaloside IV (20 mg/kg, i.p.) at 30 min after occlusion, with outcomes measured at 72 h post-reperfusion.	Brain water content↓, cerebral infarct size↓, ROS↓, MDA↓, GSH↓, TNF-α↓, NF-κB↓, IL-6↓, IL-1β↓, GPX4↓, SLC7A11↓, Nrf2↓,HO-1↓	Zhang et al. (2023)
	*Anoectochilus roxburghii* (Wall.) Lindl	Kinsenoside	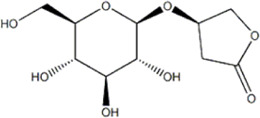	C57BL/6J mice bEnd.3 cell	*In vivo*: MCAO mice received Kinsenoside (1, 5, 10 μg, intracerebroventricular) immediately upon reperfusion after 1 h of ischemia, with outcomes measured at 72 h post-reperfusion. *In vitro*: Cells were treated with Kinsenoside (10, 15, 20 μg/mL) during the 14-h reoxygenation phase following OGD/R.	Brain water content↓, cerebral infarct size↓, ROS↓, SOD↑, Nrf2↓, HO-1↓, Occludin↑, claudin-5↑, ZO-1 ↑	Qiao et al. (2023)
	*Glycine max* (L.) Merr.	Soy Isoflavones	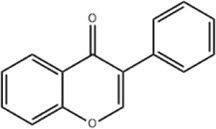	ICR rats PC12 cells	*In vivo*: BCCAO rats received oral soy isoflavones (100 mg/kg) for 5 days prior to ischemia-reperfusion, with outcomes measured at 24 h post-reperfusion. *In vitro*: PC12 cells were treated with soy isoflavones (140, 280, 560 μg/mL) during reoxygenation after OGD/R for 24 h.	Brain water content↓, cerebral infarct size↓, SOD↑, GSH↑, CAT ↑, MDA↓, LDH ↓, Nrf2↑, HO-1↑, NQO1↑, Keap1 ↓	Xue et al. (2025)
	*Apium graveolens* L. var. dulce DC., *Matricaria recutita* L., *Petroselinum crispum*	Apigenin	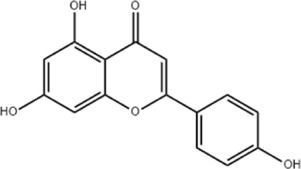	SD rat	*In vivo*: pMCAO rats were pre-treated with apigenin (30, 60, 120 mg/kg) *via* gavage for 7 days before ischemia, with outcomes measured at 24 h after pMCAO.	Brain water content↓,cerebral infarct size↓, SOD↑, GSH↑, ROS↑, MDA↓, caspase↓, Cyt c↓	Ping et al. (2024)
	*Carthamus tinctorius* L.	Hydroxysafflor yellow A	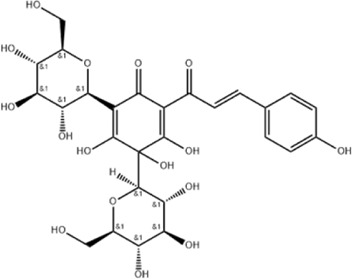	SD rat	*In vivo*: MCAO rats received intravenous HSYA (2.5, 5, 10 mg/kg) at 1 h after ischemia onset, with outcomes measured at 24 h after ischemia.	Brain water content↓, cerebral infarct size↓, iNOS↓, NO↓	Sun et al. (2013)
Anti-apoptosis	*Schisandra chinensis* (Turcz.) Baill., *Schisandra sphenanthera* Rehd. et Wils.	Schisandrin B	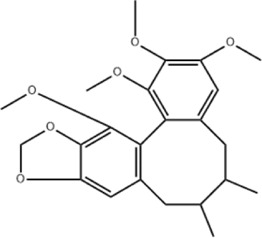	SD rat	*In vivo*: MCAO rats received intraperitoneal Sch B (50, 100 mg/kg) at 2 and 12 h after ischemia onset, with outcomes measured at 24 and 72 h after MCAO.	Brain water content↓, cerebral infarct size↓, Bax/Bcl-2↓, caspase-3↓, IL-18↓, MDA↓, NO↓, SOD↑	Hong et al. (2023)
	*Ginkgo biloba* L.	Ginkgolide B	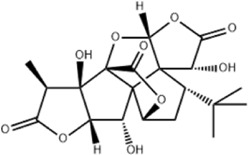	SD rat PC12 cells	*In vivo*: MCAO/R rats received oral administration of XQ-1H (7.8, 15.6, 31.2 mg/kg) starting at 4 h post-reperfusion and then every 8 h for 3 days, with outcomes measured at 72 h *In vitro*: Cells were treated with XQ-1H (1, 3, 10, 50, 100 μM) for 24 h during reoxygenation after OGD/R.	Brain water content↓, cerebral infarct size↓, bcl-2↑, bax↓	Khadankhuu et al. (2021)

**TABLE 3:** Statistics of basic experimental studies on the treatment of post-stroke cerebral edema with Chinese herbal formulae.

Effect	Formulas	Complete composition	Animal/Cell	Treatment	Targets	References
Regulating fluid metabolism and ion homeostasis	Tongxinluo	*Panax ginseng* C. A. Mey., *Hirudo nipponica* Whitman, *Buthus martensii* Karsch, *Paeonia lactiflora* Pall., *Cryptotympana pustulata* Fabricius, *Eupolyphaga sinensis* Walker, *Scolopendra subspinipes* mutilans L. Koch, *Santalum album* L., *Dalbergia odorifera* T. Chen, *Boswellia carterii* Birdw., *Ziziphus jujuba* Mill. var. spinosa (Bunge) Hu ex H. F. Chow, *Dryobalanops aromatica* Gaertn. f. (for natural Borneol)	SD mice	*In vivo*: MCAO/R rats received oral administration of Tongxinluo (1.6 g/kg/day, twice daily) for 3 days before and after ischemia, with outcomes measured at 24 h and 72 h post-reperfusion.	Brain water content↓, AQP4↓, HMGB1↓, TLR4↓, NF-κB↓	Cai et al. (2016)
	Goreisan	*Polyporus umbellatus* (Pers.) Fries, *Poria cocos* (Schw.) Wolf, *Atractylodes macrocephala* Koidz., *Alisma orientale* (Sam.) Juzep., *Cinnamomum cassia* Presl	ddY mice	*In vivo*: MCAO mice received oral administration of Goreisan (500 mg/kg, twice daily) for 5 days before ischemia, with outcomes measured at 24 h post-reperfusion.	Brain water content↓, AQP4↓	Nakano et al. (2018)
Protecting and repairing the BBB	YiQiFuMai powder injection	*Panax ginseng* C. A. Mey., *Ophiopogon japonicus* (L. f.) Ker-Gawl. *Schisandra chinensis* (Turcz.) Baill.	C57BL/6J mice	*In vivo*: MCAO mice received intraperitoneal administration of YiQiFuMai powder injection (0.336, 0.671, and 1.342 g/kg) at 1 h post-reperfusion, with outcomes measured at 24 h.	Brain water content↓, cerebral infarct size↓, leakage of EB↓, ZO-1↑, occludin↑	Cao et al. (2016)
	Tongxinluo	*Panax ginseng* C. A. Mey., *Hirudo nipponica* Whitman, *Buthus martensii* Karsch, *Paeonia lactiflora* Pall., *Cryptotympana pustulata* Fabricius, *Eupolyphaga sinensis* Walker, *Scolopendra subspinipes* mutilans L. Koch, *Santalum album* L., *Dalbergia odorifera* T. Chen, *Boswellia carterii* Birdw., *Ziziphus jujuba* Mill. var. spinosa (Bunge) Hu ex H. F. Chow, *Dryobalanops aromatica* Gaertn. f.	CD1 male mice	*In vivo*: tMCAO mice received oral administration of Tongxinluo (1 g/kg/day) in different regimens (pre-treatment for 7 days; pre-post treatment for 7 days before and 14 days after ischemia), with outcomes measured at multiple time points (1, 3, 7, 14 days) post-reperfusion.	Brain water content↓, cerebral infarct size↓, IL-6↓, IL-1β↓, TNF-α↓, MPO↓, BBB damage↓	Liu et al. (2013)
	Cerebralcare Granule	*Astragalus membranaceus* (Fisch.) Bge. *Hirudo nipponica* Whitman, *Acorus tatarinowii* Schott, *Achyranthes bidentata* Blume, *Paeonia suffruticosa* Andr., *Rheum palmatum* L., *Ligusticum chuanxiong* Hort.	SD mice	*In vivo*: MCAO rats received oral Cerebralcare Granule (0.4 g/kg or 0.8 g/kg) at 3 h post-reperfusion and then once daily for 6 days, with outcomes measured at 3 h and 6 days post-reperfusion.	Brain water content↓, cerebral infarct size↓, claudin-5↑, JAM-1↑, occludin↑, zonula↑,occluden-1↑, caveolin-1↓	Huang et al. (2012)
	Houshiheisan	*Chrysanthemum morifolium* Ramat., *Atractylodes macrocephala* Koidz., *Asarum sieboldii* Miq., *Poria cocos* (Schw.) Wolf, *Ostrea gigas* Thunberg, *Platycodon grandiflorum* (Jacq.) A. DC., *Saposhnikovia divaricata* (Turcz.) Schischk., *Panax ginseng* C. A. Mey., Alum, *Scutellaria baicalensis* Georgi, *Angelica sinensis* (Oliv.) Diels, *Zingiber officinale* Rosc., *Ligusticum chuanxiong Hort., Cinnamomum* cassia Presl.	SD mice Im-HUVECs	*In vivo*: pMCAO rats received intragastric administration of Houshiheisan (5.25 g/kg or 10.5 g/kg) at 6 h post-modeling and then once daily for 7 consecutive days, with outcomes measured at 7 days. *In vitro*: Cells were treated with HSHS medicated serum (2.5%, 5%, 10%, or 20%) for 24 h prior to and during OGD for 6 h, with outcomes measured post-OGD.	Blood vessel edema↓, SDF-1α↓, CXCR4↓, HIF-1α↑, VEGFA↑, Ang-1↑, Ang-2↓	Xiang et al. (2019)
Inhibiting neuroinflammatory cascades	Zhongfeng Xingnao prescription	*Panax ginseng* C. A. Mey., *Panax notoginseng* (Burk.) F. H. Chen, *Ligusticum chuanxiong* Hort., *Rheum palmatum* L.	SD mice	*In vivo*: ICH rats received intragastric administration of Zhongfeng Xingnao Prescription (4.5, 9, or 18 mL/kg) starting at 24 h post-modeling and then once daily for 5 consecutive days, with outcomes measured at 5 days.	Edema and vacuole conditions around the hematoma↓, p-NF-κB p65↓, NLRP3↓, Caspase1↓, IL-1β↓, TNF-α↓	Yu et al. (2025)
	QiShenYiQi	*Astragalus membranaceus* (Fisch.) Bge., *Salvia miltiorrhiza* Bunge, *Panax notoginseng* (Burk.) F. H. Chen, *Dalbergia odorifera* T. Chen.	ICR mice	*In vivo*: MCAO mice were pretreated with QSYQ (56.9, 113.8, 227.5 mg/kg) orally for 7 days before surgery (1 h ischemia/24 h reperfusion), with outcomes measured at 24 h.	Brain water content↓, cerebral infarct size↓, IFNG-γ↓, IL-1β↓,IL-6↓, TNF-α↓, NF-κB p65↓, TGF-β1↓, TLR-4↓	Wang et al. (2020)
Anti-apoptosis	Danhong injection	*Salvia miltiorrhiza* Bunge, *Carthamus tinctorius* L.	SD mice	MCAO rats received intravenous DHI (0.5, 1.0, 2.0 mL/kg) once daily for 7 days post-reperfusion, with outcomes measured at day 7.	Brain water content↓, cerebral infarct size↓, MDA↓, SOD↑, T-AOC↑, NAD ↓, pyruvate↓, HIF1α↓, PDK1↓, pPDHA1↓	Zeng et al. (2021)

^a^ The medicinal compositions of all formulas listed in this table are directly extracted and summarized from the original research articles cited in the “Reference” column.

### 4.1 Regulating fluid metabolism and ion homeostasis

Disorders of fluid metabolism and ion homeostasis are the core pathological hubs of post-stroke cerebral edema, presenting distinct processes in cerebral ischemia and intracerebral hemorrhage. Cerebral ischemia initially causes sodium and water retention in neurons due to Na^+^/K^+^-ATPase dysfunction from energy failure (Sylvain et al., 2021), forming cytotoxic edema, accompanied by abnormal polarity distribution of astrocyte AQP4 aquaporins, which exacerbates swelling (Kitchen et al., 2020; Markou et al., 2022). Intracerebral hemorrhage directly impairs microcirculation due to mechanical compression by hematoma, combined with thrombin activation and free iron from hemoglobin degradation, which damage endothelial ion transport function, triggering vasogenic edema (Wan et al., 2023). Therefore, regulating fluid metabolism and ion homeostasis may reduce the severity of post-stroke cerebral edema: for post-ischemic cerebral edema, the focus is on repairing the sodium-potassium pump and regulating the expression and distribution of aquaporin AQP4; for post-hemorrhagic cerebral edema, the emphasis is on clearing toxic substances and restoring endothelial channel function.

Regulating the expression and distribution of AQP4 is a hypothesized key mechanism for reducing astrocyte swelling and stabilizing BBB integrity. Preclinical studies suggest that several TCM interventions may act through this pathway. For instance, experimental models indicate that Tongxinluo Capsule can downregulate AQP4 expression, reduce T2WI relative signal intensity, and decrease brain water content (Cai et al., 2016). Similarly, the classic formula Wuling Powder has been shown in mice to reduce brain water content after ischemic stroke by suppressing AQP4, a mechanism that involves Mn^2+^ and Zn^2+^ ions and is consistent with its observed inhibition of AQP4-dependent permeability in MLE-12 cells (Nakano et al., 2018). In addition, emodin, a botanical metabolite exerts a protective effect on post-ischemic cerebral edema both *in vivo* and *in vitro*, inhibiting astrocyte swelling and BBB disruption mediated by AQP4 (Chen et al., 2024). Other botanical metabolites, such as cornel glycosides, which significantly reduced cerebral edema (p = 0.0018) (Wang et al., 2024b), and total steroid saponins, which also significantly reduced cerebral edema (p < 0.01) (Zhang et al., 2014), function through this pathway.

Restoring ion pump function (enhancing Na^+^/K^+^-ATPase activity) and regulating ion channels (promoting intracellular Na^+^ and Ca^2+^ efflux) are core strategies for intervening in cytotoxic edema, aiming to maintain ion homeostasis in neurons and glial cells. The botanical metabolite gastrodin, for example, has been demonstrated in rat models to reduce excitotoxicity and oxidative damage by inhibiting glutamate release, blocking calcium overload, and reducing nitric oxide production, thereby decreasing infarct and edema volumes and improving neurological function (Zeng et al., 2006).

### 4.2 Protecting and repairing the BBB

Vasogenic edema is the main type of edema in intracerebral hemorrhage and the main form of edema transformation after cerebral ischemia-reperfusion, fundamentally resulting from disrupted BBB integrity, leading to extravasation of plasma components, especially protein-rich fluids, into the brain parenchyma. In the early stage of injury, activated endothelial cells and microglia/infiltrating inflammatory cells release matrix metalloproteinases MMP-9/MMP-2, which disrupt the BBB through a continuous three-level cascade (Lu and Wen, 2024): first, degrading key components of the basement membrane, such as laminin and type IV collagen; then, exposing adhesion receptors on the endothelial cell surface, promoting further adhesion and infiltration of inflammatory cells; finally, directly hydrolyzing tight junction proteins occludin, claudin-5, and ZO-1, leading to complete disintegration of anchoring structures between endothelial cells (Wang P. et al., 2022). Extravasated plasma components accumulate in the brain tissue interstitium to form vasogenic edema, and mechanically compress the local microcirculation system, exacerbating ischemic injury, thus forming a vicious cycle where edema aggravates ischemia and ischemia further exacerbates edema (Ng et al., 2022). Therefore, targeted inhibition of matrix metalloproteinase activity and promotion of tight junction protein network reconstruction are important intervention strategies to reduce cerebral edema and improve neurological prognosis.

The key to repairing BBB tight junction structures lies in enhancing the expression, proper assembly, and localization of junction proteins (such as ZO-1, occludin, claudin-5). This is usually achieved by activating protective signaling pathways (such as HO-1, GSK-3β/β-catenin) or inhibiting destructive factors (such as caveolin-1) to reduce plasma leakage. For instance, Yiqi Fumai Powder Injection has been shown in mouse models to attenuate BBB leakage, upregulate the tight junction proteins ZO-1 and occludin (Cao et al., 2016). Similarly, Tongxinluo Capsule shares the same anti-edema mechanism in treating post-stroke cerebral edema (Liu et al., 2013). Naomao Granule alleviates BBB disruption and ischemic post-stroke cerebral edema by protecting tight junction proteins between cerebral microvascular endothelial cells, such as claudin-5, JAM-1, occludin, ZO-1, and inhibiting the expression of caveolin-1 (Huang et al., 2012). At the metabolite level, naringenin is proposed to upregulate tight junction proteins *via* the GSK-3β/β-catenin pathway (Yang et al., 2024), while curcumin (Wang et al., 2013) and baicalin (Zhu et al., 2012) have been reported to enhance junctional integrity, potentially through activating the HO-1 pathway.

Inhibiting the expression and enzymatic activity of matrix metalloproteinases (MMPs, especially MMP-9 and MMP-2) is an effective mechanism to reduce vasogenic edema. This can be achieved by blocking their upstream activation signaling pathways (such as NF-κB, ERK1/2) or directly interfering with their synthesis and activation processes, effectively protecting the basement membrane and tight junction proteins from degradation. Preclinical studies indicate that crocin protects cortical microvascular endothelial cells from ultrastructural damage after cerebral ischemia-reperfusion. These protective effects are associated with the inhibition of ERK1/2 phosphorylation, blockade of GRK2 membrane translocation, and downregulation of MMP-9 expression, which collectively contribute to the reduction of oxidative stress and vasogenic edema, conferring multi-faceted protection (Zheng et al., 2007). Dihydrocaffeic acid, a metabolite of coffee components, has been reported to inhibit the expression and activation of MMP-2 and MMP-9, exerting a protective effect on ischemia-induced neuronal injury and cerebral edema (Lee et al., 2015). Ginsenoside Rd is proposed to reduce BBB injury and edema severity by inhibiting proteasome activity, reducing IκBα degradation, blocking NF-κB activation pathway, and downregulating MMP-9 expression (Zhang et al., 2020). Tetrahydro curcumin reduces MMP-9 activity and oxidative damage by inhibiting homocysteinylation of cytochrome c (Hcy-cyto-c), thereby alleviating BBB disruption and cerebral edema (Tyagi et al., 2012).

Promoting angiogenesis and improving cerebral blood perfusion help alleviate ischemic injury and secondary edema. Houshi Heisan can promote angiogenesis through the HIF-1α/VEGF and SDF-1/CXCR4 pathways, relieve vascular edema, and reduce injury to blood vessels and neurons in the ischemic area (Xiang et al., 2019). Separately, studies suggest that the hexane extract of Uncaria rhynchophylla enhances endothelium-dependent vasodilation by activating the Akt/eNOS signaling pathway, improves perfusion in the ischemic penumbra, and may thereby contribute to the reduction of cerebral edema (Park et al., 2011).

### 4.3 Inhibiting neuroinflammatory cascades

Neuroinflammation is a key bridge connecting cytotoxic and vasogenic edema, amplifying both processes. In the acute phase of stroke, activated microglia, infiltrating neutrophils, and reactive astrocytes release various pro-edema mediators, including IL-1β, HMGB1, and MMPs (Chen et al., 2021; Wang X. et al., 2022). These factors exacerbate edema formation through multiple pathways: IL-1β and HMGB1 activate the endothelial cell NF-κB pathway, induce matrix metalloproteinase expression, and indirectly disrupt the integrity of BBB tight junction proteins; simultaneously activated complement cascades produce C3a/C5a, synergistically promoting leukocyte adhesion and inflammatory infiltration. Sustained inflammatory stimulation also disrupts the polar distribution of aquaporin AQP4, promoting the transformation of cytotoxic edema to vasogenic edema (Zheng et al., 2025). It should be noted that pro-inflammatory mediators released by reactive astrocytes in the early stage exacerbate injury, but they also participate in protection in the later stage by secreting neurotrophic factors and barrier repair proteins. Therefore, early targeted intervention in the neuroinflammatory cascade can effectively reduce cerebral edema and improve neurological prognosis.

Targeting the TLR4/NF-κB signaling pathway represents a promising strategy to reduce neuroinflammation and cerebral edema, as this pathway is activated by DAMPs to drive pro-inflammatory factor expression. Similarly, inhibition of the NLRP3 inflammasome and its upstream signals (e.g., CaMKII/NF-κB) may effectively block inflammatory cascade amplification, thereby reducing BBB injury (Cai et al., 2016). Zhongfeng Xingnao Decoction was shown to reduce inflammatory responses and the volume of perihematomal edema by regulating the activation of NLRP3 inflammasome after intracerebral hemorrhage, which is related to the CaMKII/NF-κB p65/NLRP3/GSDMD signaling axis (Yu et al., 2025). Pretreatment with Qishen Yiqi Formula in mouse models inhibited neuroinflammatory responses, protected the BBB, and reduced cerebral edema, which significantly attenuated midline shift caused by brain swelling (p < 0.001), concomitant with downregulation of IFNG-γ, IL-6, TNF-α, NF-κB p65, and TLR-4 mRNA (Wang et al., 2020). Network pharmacological analysis shows that Miaoyao Xuemaitong Capsule can alleviate intracerebral hemorrhage-induced inflammation and edema by regulating the TNFR1/NF-κB and TNFR1/MAPK signaling pathways (Zhang et al., 2021). In addition, the botanical metabolite verbenalin reduces acute inflammatory injury and cerebral edema in intracerebral hemorrhage by inhibiting TLR4 (Lai et al., 2019). Both jolkinolide B (Ma et al., 2024) and ginkgolide B (Chen et al., 2018) improve hypoxic-ischemic brain injury in newborn male rats by inhibiting NLRP3 inflammasome activation, alleviating cerebral edema. Ginkgolide C (Li et al., 2023) reduces inflammation-induced injury caused by cerebral ischemia/reperfusion by inhibiting the CD40/NF-κB pathway, improves microvascular ultrastructural characteristics, and ameliorates BBB disruption, thereby reducing cerebral edema.

Regulating the MAPK signaling pathway (such as JNK, p38) is an important approach to intervene in inflammatory mediator release and BBB disruption. Experimental studies suggest that leonurine can reduce BBB disruption *in vivo* by inhibiting hemoglobin degradation and inflammatory mediator release, a process associated with the JNK signaling pathway and the inhibition of oxyhemoglobin-induced inflammatory protein expression in BV-2 cells (Lin et al., 2016). Similarly, proanthocyanidins have been shown in rat models to exert neuroprotective effects against cerebral ischemia, which are correlated with the inhibition of the TLR4-p38-NF-κB-NLRP3 signaling pathway and a reduction in cerebral edema (Yang et al., 2020).

Modulating the activity of microglia and astrocytes offers a broader anti-inflammatory approach. The combination of jolkinolide B and ginsenoside Rg1 has been reported to reduce neuroinflammation and cerebral edema in experimental models, potentially by inhibiting miR-155-5p expression in microglia (Wang et al., 2018). Ginkgo diterpene lactones can alleviate OGD/R injury in primary astrocytes and LPS-induced inflammatory responses, reducing infarct volume and cerebral edema (Li X. et al., 2020). Methanol extracts of Glycyrrhiza uralensis and its roots regulate inflammation-related neuronal cells such as microglia and astrocytes, thereby reducing edema volume and exerting neuroprotective effects on ischemic stroke (Choi et al., 2022). Other botanical metabolite such as tetramethylpyrazine (Liao et al., 2004), paeonol (Zhao et al., 2018), and furanocoumarin dimers (Mi et al., 2021) also function through this mechanism. Other pathways, such as artemisinin, can upregulate the expression of neural cell adhesion molecule L1, inhibit inflammation and oxidative stress, and alleviate cerebral edema (Wang X. et al., 2022).

### 4.4 Anti-oxidative stress

After ischemic or hemorrhagic stroke, excessive production of reactive oxygen species and reactive nitrogen species triggers cascading oxidative damage, leading to lipid peroxidation of vascular endothelial cell membranes (Kim et al., 2021), and inducing nitration or carbonylation of key structural proteins such as tight junction proteins, thereby disrupting BBB integrity, increasing microvascular permeability, and ultimately promoting the formation of vasogenic edema (Chen S. et al., 2022). In addition, mitochondrial oxidative stress can further exacerbate neuronal apoptosis and cytotoxic edema (Panickar and Anderson, 2011). Therefore, anti-oxidative stress can indirectly exert anti-edema effects on post-stroke cerebral edema by repairing the BBB.

Activating the Nrf2/HO-1 signaling pathway is a core cellular anti-oxidative defense mechanism. Preclinical studies indicate that several TCM interventions may confer protection by enhancing the activity of endogenous antioxidant enzymes and decreasing levels of lipid peroxides. For instance, Multiple studies have demonstrated that Dracocephalum moldavica (Jia et al., 2017), lavender oil (Vakili et al., 2014), Houttuynia cordata, and the botanical metabolite oleanolic acid (Cho et al., 2009) can enhance endogenous antioxidant defense, inhibit oxidative stress, and thus reduce cerebral edema by decreasing malondialdehyde content and increasing the activities of superoxide dismutase and glutathione peroxidase. Among them, lavender oil also exerts synergistic effects by increasing VEGF expression. Intranasal administration of catalpol upregulates the expression of Nrf2 and HO-1 to reduce oxidative stress injury, increase SOD activity, decrease MDA activity, and reduce infarct volume, neurological dysfunction, and cerebral edema (Wang P. et al., 2022) The botanical metabolite astragaloside IV (Zhang et al., 2023), dendrobium nobile glycoside (Qiao et al., 2023), and soy isoflavones (Xue et al., 2025) also function through this signaling pathway. In addition, apigenin can promote DNA repair by inhibiting the PARP1/AIF pathway, reduce ROS and malondialdehyde, increase glutathione and superoxide dismutase, improve antioxidant capacity, thereby reducing infarct volume and neurological deficits, decreasing edema volume, and reducing blebbing and cell death (Ping et al., 2024).

Direct scavenging of harmful reactive nitrogen species or inhibiting their key generating enzymes can specifically reduce oxidative damage such as protein nitration, thereby alleviating cerebral edema. Hydroxysafflor yellow A reduces protein tyrosine nitration by directly scavenging peroxynitrite and inhibiting the expression of inducible nitric oxide synthase (iNOS), thereby reducing post-ischemic cerebral edema (Sun et al., 2013).

### 4.5 Anti-apoptosis

In cerebral ischemia-reperfusion injury, activation of the neuronal apoptotic pathway exacerbates cerebral edema through dual mechanisms: caspase activation driven by the mitochondrial pathway leads to caspase-3-mediated cleavage of the Na^+^/K^+^-ATPase α subunit, directly di (Cheng et al., 2020; Panickar and Anderson, 2011)srupting ion homeostasis and worsening cytotoxic edema. Meanwhile, damage-associated molecular patterns released by apoptotic neurons activate microglia, triggering cascading release of pro-inflammatory factors such as TNF-α and IL-1β, which further degrade tight junction proteins and induce vasogenic edema (Sadeghian et al., 2019).

Targeting upstream apoptotic signal hubs (PI3K/AKT, MAPK) can inhibit apoptosis. Experimental evidence suggests that Shilong Qingxue Granule (SQG) alleviates glutamate-induced neuronal calcium overload, ROS accumulation, and mitochondrial damage by inhibiting the mitochondrial apoptotic pathway and regulating the MAPK signaling pathway, thereby relieving cytotoxic edema (Hu et al., 2025). Schisandrin reduces apoptosis, inflammatory responses, and oxidative stress in MCAO rats by regulating the PI3K/AKT pathway, preventing ischemic brain injury and reducing brain water content (Hong et al., 2023). Ginkgolide B has been shown *in vivo* and *in vitro* to inhibit apoptosis by activating the PI3K/Akt pathway, suggesting a neuroprotective effect against cerebral ischemia/reperfusion injury that includes a reduction in cerebral edema (Khadankhuu et al., 2021).

Inhibiting apoptosis can be achieved by intervening in related processes such as autophagy and energy metabolism. Excessive autophagy can induce apoptotic death, and energy failure directly activates apoptosis, both of which are closely coupled with apoptosis. Danhong Injection has been demonstrated in rat models to alleviate cerebral edema and rescue neuronal injury in the ischemic penumbra. This effect is proposed to involve the regulation of intracellular energy metabolism—namely, restoring cytoplasmic glycolytic activity and relieving mitochondrial metabolic inhibition—potentially through inhibiting the activation of the PARP1/AIF and HIF1α/PDK1 signaling pathways (Zeng et al., 2021). Arctigenin improves cerebral ischemia by inhibiting neuronal apoptosis and AMPK/mTOR-mediated autophagy, reducing cerebral edema and improving neurological function scores (Yang et al., 2021).

### 4.6 Other potential mechanisms

Focusing on metabolic homeostasis repair, regulating key metabolic pathways such as membrane components and energy metabolism can reduce glial cell edema. A study using metabolomics demonstrated that Longxue Tongluo Capsule can normalize metabolic disorders and reduce neuroglial interstitial edema by regulating metabolic pathways involving glycerophospholipid metabolism, glycosylphosphatidylinositol anchor biosynthesis, nicotinate and nicotinamide metabolism, and sphingolipid metabolism (Sun et al., 2023). Improving cerebral microcirculation, anti-platelet aggregation, and anti-thrombosis can reduce neuronal necrosis and glial cell edema caused by ischemia. Supercritical CO_2_ extract of Salvia miltiorrhiza (SCED) improves microcirculation by inhibiting platelet PLC/PKC signaling pathway activation, blocking thromboxane A2 release, and inhibiting platelet aggregation, thereby improving cerebral blood perfusion and reducing cerebral ischemic injury and edema (Fei et al., 2017).

## 5 Current limitations and future perspectives

### 5.1 Defects in clinical trial design

Although multiple clinical studies have demonstrated the improvement effect of Chinese botanical drug preparations, Chinese botanical drug, and their metabolites on post-stroke cerebral edema, there remains a problem of insufficient sample size. Moreover, the included clinical studies exhibited certain heterogeneity in experimental design, with some lacking detailed double-blind procedures, which may increase the risk of bias. Some studies did not perform formal sample size calculations or power analyses, potentially limiting the statistical reliability of their findings.

Furthermore, most available publications tend to report positive or favorable outcomes, while studies with negative or inconclusive findings are rarely accessible, particularly within Chinese-language databases. This publication and selection bias may lead to an overestimation of the therapeutic efficacy of Chinese botanical drugs. Another critical issue identified in the reviewed clinical trials is the lack of standardized safety reporting. Adverse event reporting is often inconsistent or inadequately detailed, which hinders the comprehensive assessment of the safety profile of Chinese botanical drugs in clinical settings. This gap in safety reporting, combined with the aforementioned methodological issues, further diminishes the overall reliability of the evidence.

Future investigations should prioritize well-designed randomized controlled trials (RCTs) with adequate sample sizes and standardized outcome measures to enhance the robustness and reproducibility of clinical evidence. In particular, studies should adhere to rigorous adverse event reporting standards to ensure comprehensive safety evaluation. In addition, the differential effects of TCM in different stages of cerebral edema, i.e., the acute phase (0–72 h) and the recovery phase (7–14 days), need further exploration.

### 5.2 Insufficient translation from basic research to clinical practice

Basic studies have revealed multiple mechanisms through which TCM may alleviate post-stroke cerebral edema. However, despite these mechanistic advances, effective clinical translation remains limited. Many preclinical findings have not been systematically verified in humans, leading to uncertainties in dose–response relationships, pharmacodynamic stability, and safety under complex clinical conditions (Li et al., 2022; Sun et al., 2019).

Key barriers include inconsistent dose conversion between animal and human studies, variability in pharmacokinetics among multi-component botanical formulations, and the absence of unified regulatory and quality-assessment frameworks. Furthermore, poor bioavailability and limited blood–brain barrier permeability of metabolites often compromise therapeutic efficacy (Sanchez-Martinez et al., 2022).

Future research should prioritize dose optimization, formulation improvement to enhance bioavailability, and pharmacokinetic–pharmacodynamic modeling (Dewi et al., 2022; Xie et al., 2025). Integrating TCM-based evidence into standardized and evidence-based evaluation systems will be essential to improve credibility and facilitate clinical adoption (Li R. et al., 2024).

### 5.3 Difficulties in analyzing components of Chinese medicines

In basic research, TCM, especially botanical formulas, has extremely complex compositions: a single Chinese medicinal material may contain dozens to hundreds of active metabolites, while formulas are combinations of multiple medicinal materials, leading to superimposed metabolites. This complexity enables them to exert therapeutic effects through multiple mechanisms, but precise analysis of specific molecular targets is challenging. The optimal effective doses and proportions of different active components in compound formulas, as well as the pathways through which different components synergistically function, require in-depth research.

### 5.4 Challenges in quality control of botanical drug

Contamination and adulteration of TCM raw materials can cause severe adverse reactions. A systematic review including 26 cases of botanical drug contamination and adulteration pointed out that the most common herbal contaminants include dust, pollen, insects, rodents, parasites, microorganisms, fungi, molds, toxins, pesticides, and toxic heavy metals. Adulteration and contamination can cause severe adverse reactions such as arsenic, lead, or mercury poisoning, hepatorenal syndrome, nephrotoxicity, metabolic acidosis, renal or liver failure, cerebral edema, and coma (Posadzki et al., 2013). Contamination and adulteration of TCM are core issues threatening medication safety, which need to be addressed through multiple dimensions such as technological innovation, strengthened supervision, and international cooperation to unify standards.

In recent years, several pharmacopeial and international standards have been established to improve quality assurance in botanical drug production. The Chinese Pharmacopoeia (2020 Edition) and WHO Monographs on Selected Medicinal Plants specify detailed requirements for botanical identification, permissible levels of heavy metals, pesticide residues, and microbial contamination (Leong et al., 2020). In parallel, modern analytical techniques such as high-performance liquid chromatography, liquid chromatography–mass spectrometry, gas chromatography, and emerging molecular tools like DNA barcoding and metabarcoding have greatly improved the accuracy of species authentication and detection of adulterants (Bogusz et al., 2006; Techen et al., 2014). Integration of these pharmacopeial standards and analytical methods provides a practical and effective framework for ensuring the safety, consistency, and authenticity of TCM products (Wang et al., 2023).

Future research should focus on the toxic mechanisms and risk thresholds of contaminants to provide a basis for formulating scientific limit standards.

## 6 Conclusion

This review has synthesized evidence on the efficacy, safety, and potential mechanisms of selected TCM interventions for post-stroke cerebral edema. Clinical evidence, primarily from preliminary studies, suggests that specific TCM formulations can reduce cerebral edema volume and improve neurological function with a generally acceptable safety profile. The preclinical findings summarized herein indicate that these benefits may be mediated by multi-target effects, including the regulation of ion homeostasis and aquaporins, inhibition of neuroinflammatory cascades, protection of the blood-brain barrier, and mitigation of oxidative stress and apoptosis.

While osmotic agents and surgery remain the standard of care, the profiled TCM interventions hold promise as adjunctive therapies due to their potential to simultaneously target multiple interconnected pathways in the edema pathological network. However, this very complexity necessitates future research that prioritizes rigorous, large-scale clinical trials to confirm efficacy, standardized safety monitoring, and mechanistic studies to establish causal relationships in humans and identify the key active constituents. Overcoming these challenges will be crucial to translate these promising preclinical findings into validated therapeutic strategies.

## Glossary

ACI: Acute Cerebral Infarction
ADL: Activities of Daily Living
ADMA: Asymmetric Dimethylarginine
AIF: Apoptosis-Inducing Factor
AIS: Acute Ischemic Stroke
Ang-1: Angiopoietin-1
AQP4: Aquaporin 4
BAI: Barthel Index
BBB: Blood-Brain Barrier
BCCAO: Bilateral Common Carotid Artery Occlusion
BDNF: Brain-Derived Neurotrophic Factor
bid: bis in die (twice daily)
BMVECs: Brain Microvascular Endothelial Cells
CaMKII: Calcium/Calmodulin-Dependent Protein Kinase II
CAT: Catalase
CBS: Cystathionine Beta-Synthase
CD40: Cluster of Differentiation 40
CD44: Cluster of Differentiation 44
CD163: Cluster of Differentiation 163
CNKI: China National Knowledge Infrastructure
CXCR4: C-X-C Chemokine Receptor Type 4
EB: Evans Blue
EGFR: Epidermal Growth Factor Receptor
ERK: Extracellular Signal-Regulated Kinase
ESS: European Stroke Scale
ET: Endothelin
FIB: Fibrinogen
GAPDH: Glyceraldehyde 3-Phosphate Dehydrogenase
GCS: Glasgow Coma Scale
GSDMD: Gasdermin D
GSH: Glutathione
GSH-px: Glutathione Peroxidase
GPI: Glycosylphosphatidylinositol
Hcy: Homocysteine
HIF-1α: Hypoxia-Inducible Factor 1-alpha
HMBG1: High-Mobility Group Box 1
HO-1: Heme Oxygenase-1
HSP: Heat Shock Protein
hs-CRP: High-Sensitivity C-Reactive Protein
ICH: Intracerebral Hemorrhage
ICAM-1: Intercellular Adhesion Molecule 1
IFN-γ: Interferon Gamma
iNOS: Inducible Nitric Oxide Synthase
IL: Interleukin
ivgtt: intravenous drip
JAM-1: Junctional Adhesion Molecule 1
JNK: c-Jun N-Terminal Kinase
Keap1: Kelch-like ECH-Associated Protein 1
LC3-II: Microtubule-Associated Protein 1A/1B-Light Chain 3-II
LDH: Lactate Dehydrogenase
LPS: Lipopolysaccharide
MABP: Mean Arterial Blood Pressure
MCAO: Middle Cerebral Artery Occlusion
MBP: Myelin Basic Protein
MDA: Malondialdehyde
miR-155-5p: MicroRNA-155-5p
MMP: Matrix Metalloproteinase
MPO: Myeloperoxidase
NGF: Nerve Growth Factor
NIHSS: National Institutes of Health Stroke Scale
NO: Nitric Oxide
NQO1: NAD(P)H Quinone Dehydrogenase 1
Nrf2: Nuclear Factor Erythroid 2–Related Factor 2
NSE: Neuron-Specific Enolase
OCD/R: Oxygen-Glucose Deprivation/Reoxygenation
p-AKT: Phosphorylated AKT
PDK1: Pyruvate Dehydrogenase Kinase 1
PI3K: Phosphoinositide 3-Kinase
PKC: Protein Kinase C
po: per os (oral administration)
PRO: Procalcitonin
q4h: quaque 4 hora (every 4 hours)
qd: quaque die (once daily)
RBMEC: Rat Brain Microvascular Endothelial Cells
RCT: Randomized Controlled Trial
ROS: Reactive Oxygen Species
S-100β: S100 Calcium-Binding Protein Beta
SDF-1α: Stromal Cell-Derived Factor 1-alpha
SLC7A11: Solute Carrier Family 7 Member 11
T-AOC: Total Antioxidant Capacity
TCM: Traditional Chinese Medicine
TGF-β1: Transforming Growth Factor Beta 1
TJK: Tight Junction Protein
TLR4: Toll-Like Receptor 4
TNF-α: Tumor Necrosis Factor Alpha
tid: ter in die (three times daily)
VEGFA: Vascular Endothelial Growth Factor A
VEGFR2: Vascular Endothelial Growth Factor Receptor 2
VCAM-1: Vascular Cell Adhesion Molecule 1
ZO-1: Zonula Occludens-1
β2-MG: Beta-2-Microglobulin

## References

[B1] Bai X. Zheng E. Tong L. Liu Y. Li X. Yang H. (2024). Angong Niuhuang Wan inhibit ferroptosis on ischemic and hemorrhagic stroke by activating PPARγ/AKT/GPX4 pathway. J. Ethnopharmacol. 321, 117438. 10.1016/j.jep.2023.117438 37984544

[B2] Bogusz M. J. Hassan H. Al-Enazi E. Ibrahim Z. Al-Tufail M. (2006). Application of LC-ESI-MS-MS for detection of synthetic adulterants in herbal remedies. J. Pharm. Biomed. Anal. 41 (2), 554–564. 10.1016/j.jpba.2005.12.015 16427237

[B3] Cai M. Yu Z. Wang L. Song X. Zhang J. Zhang Z. (2016). Tongxinluo reduces brain edema and inhibits post-ischemic inflammation after middle cerebral artery occlusion in rats. J. Ethnopharmacol. 181, 136–145. 10.1016/j.jep.2016.01.026 26805468

[B4] Cao G. Ye X. Xu Y. Yin M. Chen H. Kou J. (2016). YiQiFuMai powder injection ameliorates blood-brain barrier dysfunction and brain edema after focal cerebral ischemia-reperfusion injury in mice. Drug Des. Devel Ther. 10, 315–325. 10.2147/DDDT.S96818 26834461 PMC4716731

[B5] Chen A. Xu Y. Yuan J. (2018). Ginkgolide b ameliorates NLRP3 inflammasome activation after hypoxic-ischemic brain injury in the neonatal male rat. Int. J. Dev. Neurosci. 69, 106–111. 10.1016/j.ijdevneu.2018.07.004 30030129

[B6] Chen Z. Z. Gong X. Guo Q. Zhao H. Wang L. (2019). Bu yang huan wu decoction prevents reperfusion injury following ischemic stroke in rats *via* inhibition of HIF-1 alpha, VEGF and promotion beta-ENaC expression. J. Ethnopharmacol. 228, 70–81. 10.1016/j.jep.2018.09.017 30218809

[B7] Chen Y. Chen S. Chang J. Wei J. Feng M. Wang R. (2021). Perihematomal edema after intracerebral hemorrhage: an update on pathogenesis, risk factors, and therapeutic advances. Front. Immunol. 12, 740632. 10.3389/fimmu.2021.740632 34737745 PMC8560684

[B8] Chen Y. Y. Gong Z. C. Zhang M. M. Huang Z. H. (2024). Brain-targeting emodin mitigates ischemic stroke *via* inhibiting AQP4-mediated swelling and neuroinflammation. Transl. Stroke Res. 15 (4), 818–830. 10.1007/s12975-023-01170-4 37380800

[B9] Chen H. Luo Y. Tsoi B. Gu B. Qi S. Shen J. (2022). Angong niuhuang wan reduces hemorrhagic transformation and mortality in ischemic stroke rats with delayed thrombolysis: involvement of peroxynitrite-mediated MMP-9 activation. Chin. Med. 17 (1), 51. 10.1186/s13020-022-00595-7 35477576 PMC9044615

[B10] Chen S. Li L. Peng C. Bian C. Ocak P. E. Zhang J. H. (2022). Targeting oxidative stress and inflammatory response for blood-brain barrier protection in intracerebral hemorrhage. Antioxid. Redox Signal. 37 (1-3), 115–134. 10.1089/ars.2021.0072 35383484

[B11] Cheng Y. Li S. Liu Y. Li J. Chen Y. Zhao H. (2020). Treatment of brain edema by wogonoside is associated with inhibition of neuronal apoptosis and SIRT1 activation in rats. Med. Sci. Monit. 26, e921250. 10.12659/MSM.921250 32221271 PMC7133416

[B12] Cho S. O. Ban J. Y. Kim J. Y. Ju H. S. Lee I. S. Song K. S. (2009). Anti-ischemic activities of aralia cordata and its active component, oleanolic acid. Arch. Pharm. Res. 32 (6), 923–932. 10.1007/s12272-009-1615-1 19557371

[B13] Choi M. Lim C. Lee B. K. Cho S. (2022). Amelioration of brain damage after treatment with the methanolic extract of glycyrrhizae radix et rhizoma in mice. Pharmaceutics 14 (12), 2776. 10.3390/pharmaceutics14122776 36559268 PMC9781260

[B14] Cook A. M. Morgan J. G. Hawryluk G. Mailloux P. Mclaughlin D. Papangelou A. (2020). Guidelines for the acute treatment of cerebral edema in neurocritical care patients. Neurocrit. Care 32 (3), 647–666. 10.1007/s12028-020-00959-7 32227294 PMC7272487

[B15] Cooper D. J. Rosenfeld J. V. Murray L. Arabi Y. M. Davies A. R. D’Urso P. (2011). Decompressive craniectomy in diffuse traumatic brain injury. N. Engl. J. Med. 364 (16), 1493–1502. 10.1056/NEJMoa1102077 21434843

[B16] Dewi M. K. Chaerunisaa A. Y. Muhaimin M. Joni I. M. (2022). Improved activity of herbal medicines through nanotechnology. Nanomaterials 12 (22), 4073. 10.3390/nano12224073 36432358 PMC9695685

[B17] Fan J. H. (2019). Efficacy observation of “tongfu method” (purging fu-organs method) in the treatment of cerebral edema after cerebral hemorrhage of phlegm-heat and fu-organ excess type. J. Clin. Ration. Drug Use 12 (13), 83–84. 10.15887/j.cnki.13-1389/r.2019.13.046

[B18] Fang J. Wang Z. Miao C. Y. (2023). Angiogenesis after ischemic stroke. Acta Pharmacol. Sin. 44 (7), 1305–1321. 10.1038/s41401-023-01061-2 36829053 PMC10310733

[B19] Fei Y. X. Wang S. Q. Yang L. J. Qiu Y. Y. Li Y. Z. Liu W. Y. (2017). Salvia miltiorrhiza bunge (danshen) extract attenuates permanent cerebral ischemia through inhibiting platelet activation in rats. J. Ethnopharmacol. 207, 57–66. 10.1016/j.jep.2017.06.023 28645780

[B20] Feng Y. M. Yang H. (2015). Observation on the awakening effect of angong niuhuang wan in the treatment of comatose patients with acute cerebral infarction. Chin. J. Exp. Traditional Med. Formulae 21 (06), 179–182. 10.13422/j.cnki.syfjx.2015060179

[B21] Fu Q. H. Yang H. J. Xie J. Zhou B. (2019). Efficacy observation of changpu yujin decoction on cerebral edema after craniotomy for acute intracerebral hemorrhage. Med. J. Natl. Defending Forces Southwest China 29 (05), 611–613. 10.3969/j.issn.1004-0188.2019.05.037

[B22] Gao J. Y. Shi D. L. Gao X. L. Liu T. F. Wang L. W. Cao B. (2020). Effect of huoxue ditan decoction combined with hyperbaric oxygen on neurological function recovery speed and hemodynamic level in patients with hypertensive intracerebral hemorrhage. Chin. Archives Traditional Chin. Med. 38 (09), 213–216. 10.13193/j.issn.1673-7717.2020.09.054

[B23] Hatakeyama M. Ninomiya I. Kanazawa M. (2020). Angiogenesis and neuronal remodeling after ischemic stroke. Neural Regen. Res. 15 (1), 16–19. 10.4103/1673-5374.264442 31535636 PMC6862417

[B24] He D. Liu Q. R. Zhao J. X. Dong Q. Zhang R. L. (2002). Efficacy observation of xueshuantong in the treatment of acute intracerebral hemorrhage. Chin. J. Integr. Traditional West. Med. Crit. Care (01), 27–29. 10.3321/j.issn:1008-9691.2002.01.007

[B25] Hong Q. L. Ding Y. H. Chen J. Y. Shi S. S. Liang R. S. Tu X. K. (2023). Schisandrin b protects against ischemic brain damage by regulating PI3k/AKT signaling in rats. Chin. J. Integr. Med. 29 (10), 885–894. 10.1007/s11655-023-3596-1 37357242

[B26] Hou D. W. Zhu Y. J. Zhang D. (2022). Effect observation of buyang huanwu decoction combined with ultra-early urokinase administration in patients with hypertensive intracerebral hemorrhage after minimally invasive surgery. Sichuan J. Traditional Chin. Med. 40 (09), 124–127. 10.3969/j.issn.1000-3649.2022.9.sczy202209037

[B27] Hu N. Cheng Z. Cao Y. Li X. Bai F. Wang J. (2025). Therapeutic potential of shilong qingxue granule and its extract against glutamate induced neural injury: insights from *in vivo* and *in vitro* models. J. Ethnopharmacol. 342, 119396. 10.1016/j.jep.2025.119396 39848417

[B28] Huang P. Zhou C. M. Liu Y. Y. Hu B. H. Chang X. Zhao X. R. (2012). Cerebralcare granule(r) attenuates blood-brain barrier disruption after middle cerebral artery occlusion in rats. Exp. Neurol. 237 (2), 453–463. 10.1016/j.expneurol.2012.07.017 22868201

[B29] Jia X. Q. Liu J. C. (2007). Effect of buyang huanwu decoction on β2-microglobulin (β2-mg) and cerebral edema in patients with acute cerebral infarction. Chin. J. Basic Med. Traditional Chin. Med. 7, 534–535. 10.3969/j.issn.1006-3250.2007.07.022

[B30] Jia J. X. Zhang Y. Wang Z. L. Yan X. S. Jin M. Huo D. S. (2017). The inhibitory effects of dracocephalum moldavica l. (DML) on rat cerebral ischemia reperfusion injury. J. Toxicol. Env. Health Part A 80 (22), 1206–1211. 10.1080/15287394.2017.1367139 28876179

[B31] Jiang Q. M. Yu S. Dong X. F. Wang H. S. Hou J. Huang Z. C. (2022). Predictors and dynamic nomogram to determine the individual risk of malignant brain edema after endovascular thrombectomy in acute ischemic stroke. J. Clin. Neurol. 18 (3), 298–307. 10.3988/jcn.2022.18.3.298 35196752 PMC9163945

[B32] Jiao J. Xu S. N. (2015). Clinical study on lingyang gouteng decoction (modified according to syndrome differentiation) in the treatment of elderly patients with intracerebral hemorrhage. Chin. J. Pract. Nerv. Dis. 18 (07), 21–23. 10.3969/j.issn.1673-5110.2015.07.011

[B33] Khadankhuu B. Fei Y. Li X. Fang W. Li Y. (2021). 10-o-(n n-dimethylaminoethyl)-ginkgolide b methane-sulfonate (XQ-1h) ameliorates cerebral ischemia *via* suppressing neuronal apoptosis. J. Stroke Cerebrovasc. Dis. 30 (9), 105987. 10.1016/j.jstrokecerebrovasdis.2021.105987 34273708

[B34] Kim M. Byun J. Chung Y. Lee S. U. Park J. E. Park W. (2021). Reactive oxygen species scavenger in acute intracerebral hemorrhage patients: a multicenter, randomized controlled trial. Stroke 52 (4), 1172–1181. 10.1161/STROKEAHA.120.032266 33626901

[B35] Kitchen P. Salman M. M. Halsey A. M. Clarke-Bland C. Macdonald J. A. Ishida H. (2020). Targeting aquaporin-4 subcellular localization to treat central nervous system edema. Cell 181 (4), 784–799. 10.1016/j.cell.2020.03.037 32413299 PMC7242911

[B36] Lai J. N. Tang J. L. Wang J. D. (2013). Observational studies on evaluating the safety and adverse effects of traditional Chinese medicine. Evid.-Based Complement. Altern. Med. 2013, 697893. 10.1155/2013/697893 24159351 PMC3789390

[B37] Lai X. Xiong Y. Zhou J. Yang F. Peng J. Chen L. (2019). Verbascoside attenuates acute inflammatory injury in experimental cerebral hemorrhage by suppressing TLR4. Biochem. Biophys. Res. Commun. 519 (4), 721–726. 10.1016/j.bbrc.2019.09.057 31543344

[B38] Lee K. Lee B. J. Bu Y. (2015). Protective effects of dihydrocaffeic acid, a coffee component metabolite, on a focal cerebral ischemia rat model. Molecules 20 (7), 11930–11940. 10.3390/molecules200711930 26133759 PMC6331881

[B39] Leong F. Hua X. Wang M. Chen T. Song Y. Tu P. (2020). Quality standard of traditional Chinese medicines: comparison between European pharmacopoeia and Chinese pharmacopoeia and recent advances. Chin. Med. 15, 76. 10.1186/s13020-020-00357-3 32742301 PMC7388521

[B40] Li C. Jia W. W. Yang J. L. Cheng C. Olaleye O. E. (2022). Multi-compound and drug-combination pharmacokinetic research on Chinese herbal medicines. Acta Pharmacol. Sin. 43 (12), 3080–3095. 10.1038/s41401-022-00983-7 36114271 PMC9483253

[B41] Li B. Zhang B. Li Z. Li S. Li J. Wang A. (2023). Ginkgolide C attenuates cerebral ischemia/reperfusion-induced inflammatory impairments by suppressing CD40/NF-κB pathway. J. Ethnopharmacol. 312, 116537. 10.1016/j.jep.2023.116537 37094696

[B42] Li P. Wang S. Chen Y. (2024). Use of real-world evidence in regulatory decisions for traditional Chinese medicine: current status and future directions. Ther. Innov. Regul. Sci. 58 (1), 34–41. 10.1007/s43441-023-00588-0 37996768 PMC10764529

[B43] Li R. Song M. Zheng Y. Zhang J. Zhang S. Fan X. (2024). Naoxueshu oral liquid promotes hematoma absorption by targeting CD36 in M2 microglia *via* TLR4/MyD88/NF-κB signaling pathway in rats with intracerebral hemorrhage. J. Ethnopharmacol. 319 (Pt 1), 117116. 10.1016/j.jep.2023.117116 37659762

[B44] Li X. Huang L. Liu G. Fan W. Li B. Liu R. (2020). Ginkgo diterpene lactones inhibit cerebral ischemia/reperfusion induced inflammatory response in astrocytes *via* TLR4/NF-κB pathway in rats. J. Ethnopharmacol. 249, 112365. 10.1016/j.jep.2019.112365 31678414

[B45] Li Z. Xiao G. Lyu M. Wang Y. He S. Du H. (2020). Shuxuening injection facilitates neurofunctional recovery *via* down-regulation of g-CSF-mediated granulocyte adhesion and diapedesis pathway in a subacute stroke mouse model. Biomed. Pharmacother. 127, 110213. 10.1016/j.biopha.2020.110213 32417690

[B46] Liang Z. K. Yang Y. L. (2012). Clinical observation of shuxuetong injection in the treatment of hypertensive intracerebral hemorrhage. Clin. Med. 32 (01), 57–59. 10.3969/j.issn.1003-3548.2012.01.033

[B47] Liao S. L. Kao T. K. Chen W. Y. Lin Y. S. Chen S. Y. Raung S. L. (2004). Tetramethylpyrazine reduces ischemic brain injury in rats. Neurosci. Lett. 372 (1-2), 40–45. 10.1016/j.neulet.2004.09.013 15531085

[B48] Lin S. Wu J. Guo W. Zhu Y. (2016). Effects of leonurine on intracerebral haemorrhage by attenuation of perihematomal edema and neuroinflammation *via* the JNK pathway. Pharmazie 71 (11), 644–650. 10.1691/ph.2016.6692 29441969

[B49] Lin W. Hou J. Han T. Zheng L. Liang H. Zhou X. (2022). Efficacy and safety of traditional Chinese medicine for intracranial hemorrhage by promoting blood circulation and removing blood stasis: a systematic review and meta-analysis of randomized controlled trials. Front. Pharmacol. 13, 942657. 10.3389/fphar.2022.942657 36249750 PMC9553997

[B50] Liu Y. Tang G. H. Sun Y. H. Lin X. J. Wei C. Yang G. Y. (2013). The protective role of tongxinluo on blood-brain barrier after ischemia-reperfusion brain injury. J. Ethnopharmacol. 148 (2), 632–639. 10.1016/j.jep.2013.05.018 23707212

[B51] Liu B. T. Li Q. Sun K. Pan C. S. Huo X. M. Huang P. (2024). Angong niuhuang wan ameliorates LPS-induced cerebrovascular edema by inhibiting blood‒brain barrier leakage and promoting the membrane expression of AQP4. Front. Pharmacol. 15, 1421635. 10.3389/fphar.2024.1421635 39148543 PMC11324430

[B52] Lu W. Wen J. (2024). Crosstalk among glial cells in the blood-brain barrier injury after ischemic stroke. Mol. Neurobiol. 61 (9), 6161–6174. 10.1007/s12035-024-03939-6 38279077

[B53] Lyu J. Xie Y. Sun M. Zhang L. (2020). Efficacy and safety of xueshuantong injection on acute cerebral infarction: clinical evidence and GRADE assessment. Front. Pharmacol. 11, 822. 10.3389/fphar.2020.00822 32714181 PMC7345308

[B54] Ma Y. Hu X. Shen S. Pan D. (2024). Geniposide ameliorates brain injury in mice with intracerebral hemorrhage by inhibiting NF-κB signaling. Neurol. Res. 46 (4), 346–355. 10.1080/01616412.2024.2321014 38402902

[B55] Markou A. Unger L. Abir-Awan M. Saadallah A. Halsey A. Balklava Z. (2022). Molecular mechanisms governing aquaporin relocalisation. Biochim. Biophys. Acta-Biomembr. 1864 (4), 183853. 10.1016/j.bbamem.2021.183853 34973181 PMC8825993

[B56] Mei L. Fengqun M. Xiaozhuo L. Qing W. Mingming F. Zhengyao Z. (2022). Effect western medicines combined with nao-xue-shu in patients with hypertensive intracerebral hemorrhage: a network meta-analysis. Front. Pharmacol. 13, 892904. 10.3389/fphar.2022.892904 35784744 PMC9240398

[B57] Mi Y. Jiao K. Xu J. K. Wei K. Liu J. Y. Meng Q. Q. (2021). Kellerin from ferula sinkiangensis exerts neuroprotective effects after focal cerebral ischemia in rats by inhibiting microglia-mediated inflammatory responses. J. Ethnopharmacol. 269, 113718. 10.1016/j.jep.2020.113718 33352239

[B58] Nakano T. Nishigami C. Irie K. Shigemori Y. Sano K. Yamashita Y. (2018). Goreisan prevents brain edema after cerebral ischemic stroke by inhibiting aquaporin 4 upregulation in mice. J. Stroke Cerebrovasc. Dis. 27 (3), 758–763. 10.1016/j.jstrokecerebrovasdis.2017.10.010 29153303

[B59] Nawabi J. Flottmann F. Hanning U. Bechstein M. Schon G. Kemmling A. (2019). Futile recanalization with poor clinical outcome is associated with increased edema volume after ischemic stroke. Invest. Radiol. 54 (5), 282–287. 10.1097/RLI.0000000000000539 30562271

[B60] Ng F. C. Churilov L. Yassi N. Kleinig T. J. Thijs V. Wu T. Y. (2022). Microvascular dysfunction in blood-brain barrier disruption and hypoperfusion within the infarct posttreatment are associated with cerebral edema. Stroke 53 (5), 1597–1605. 10.1161/STROKEAHA.121.036104 34937423

[B61] Pan Y. W. Wu D. P. Liang H. F. Tang G. Y. Fan C. L. Shi L. (2022). Total saponins of panax notoginseng activate akt/mTOR pathway and exhibit neuroprotection *in vitro* and *in vivo* against ischemic damage. Chin. J. Integr. Med. 28 (5), 410–418. 10.1007/s11655-021-3454-y 34581940

[B62] Pan Y. Nie L. Chen W. Guan D. Li Y. Yang C. (2025). Buyang Huanwu Decoction prevents hemorrhagic transformation after delayed t-PA infusion *via* inhibiting NLRP3 inflammasome/pyroptosis associated with microglial PGC-1α. J. Ethnopharmacol. 340, 119275. 10.1016/j.jep.2024.119275 39710159

[B63] Panickar K. S. Anderson R. A. (2011). Effect of polyphenols on oxidative stress and mitochondrial dysfunction in neuronal death and brain edema in cerebral ischemia. Int. J. Mol. Sci. 12 (11), 8181–8207. 10.3390/ijms12118181 22174658 PMC3233464

[B64] Papagianni M. Tziomalos K. Kostaki S. Angelopoulou S. M. Christou K. Bouziana S. D. (2018). Treatment with mannitol is associated with increased risk for in-hospital mortality in patients with acute ischemic stroke and cerebral edema. Am. J. Cardiovasc. Drugs 18 (5), 397–403. 10.1007/s40256-018-0285-0 29845546

[B65] Park S. H. Kim J. H. Park S. J. Bae S. S. Choi Y. W. Hong J. W. (2011). Protective effect of hexane extracts of *Uncaria sinensis* against photothrombotic ischemic injury in mice. J. Ethnopharmacol. 138 (3), 774–779. 10.1016/j.jep.2011.10.026 22051882

[B66] Ping N. Zuo K. Cai J. Rong C. Yu Z. Zhang X. (2024). Apigenin protects against ischemic stroke by increasing DNA repair. Front. Pharmacol. 15, 1362301. 10.3389/fphar.2024.1362301 38746012 PMC11091408

[B67] Posadzki P. Watson L. Ernst E. (2013). Contamination and adulteration of herbal medicinal products (HMPs): an overview of systematic reviews. Eur. J. Clin. Pharmacol. 69 (3), 295–307. 10.1007/s00228-012-1353-z 22843016

[B68] Qiao N. An Z. Fu Z. Chen X. Tong Q. Zhang Y. (2023). Kinsenoside alleviates oxidative stress-induced blood-brain barrier dysfunction *via* promoting nrf2/HO-1 pathway in ischemic stroke. Eur. J. Pharmacol. 949, 175717. 10.1016/j.ejphar.2023.175717 37054938

[B69] Sadeghian N. Shadman J. Moradi A. Ghasem G. M. Panahpour H. (2019). Calcitriol protects the blood-brain barrier integrity against ischemic stroke and reduces vasogenic brain edema *via* antioxidant and antiapoptotic actions in rats. Brain Res. Bull. 150, 281–289. 10.1016/j.brainresbull.2019.06.010 31220552

[B70] Sanchez-Martinez J. D. Valdes A. Gallego R. Suarez-Montenegro Z. J. Alarcon M. Ibanez E. (2022). Blood-brain barrier permeability study of potential neuroprotective compounds recovered from plants and agri-food by-products. Front. Nutr. 9, 924596. 10.3389/fnut.2022.924596 35782945 PMC9243654

[B71] Shang L. Wang X. Sun H. Wei W. Sun Y. Cai G. (2022). Effects of the triple therapy of carnosine glycoside, edaravone, and xueshuantong in hemorrhagic cerebral infarction. Am. J. Transl. Res. 14 (2), 1024–1033. Available online at: https://pubmed.ncbi.nlm.nih.gov/35273704/ 35273704 PMC8902570

[B72] Shang J. Huang G. Wang B. Wang J. Wei W. Cui Y. (2025). Shuxuetong injection inhibits pyroptosis in acute ischemic stroke *via* CD44/NLRP3/GSDMD signal. J. Ethnopharmacol. 345, 119618. 10.1016/j.jep.2025.119618 40074097

[B73] Song J. Nie Y. Wang P. Lu H. Gao L. (2021). Naoxueshu relieves hematoma after clot removal in acute spontaneous intracerebral hemorrhage. Brain Behav. 11 (1), e01957. 10.1002/brb3.1957 33274855 PMC7821564

[B74] Song J. Nie Y. Qin X. Wang P. Lu H. Gao L. (2022). Efficacy of naoxueshu in acute spontaneous intracerebral hemorrhage: a multicenter observational study. Neurol. Sci. 43 (3), 1885–1891. 10.1007/s10072-021-05582-8 34532772 PMC8860792

[B75] Sun L. Yang L. Fu Y. Han J. Xu Y. Liang H. (2013). Capacity of HSYA to inhibit nitrotyrosine formation induced by focal ischemic brain injury. Nitric Oxide-Biol. Chem. 35, 144–151. 10.1016/j.niox.2013.10.002 24126016

[B76] Sun S. Wang Y. Wu A. Ding Z. Liu X. (2019). Influence factors of the pharmacokinetics of herbal resourced compounds in clinical practice. Evid.-Based Complement. Altern. Med. 2019, 1983780. 10.1155/2019/1983780 30949215 PMC6425497

[B77] Sun W. Zhang L. Fang Z. Han L. Wang Q. Leng Y. (2022). Shuxuetong injection and its peptides enhance angiogenesis after hindlimb ischemia by activating the MYPT1/LIMK1/cofilin pathway. J. Ethnopharmacol. 292, 115166. 10.1016/j.jep.2022.115166 35248678

[B78] Sun J. Chen X. Wang Y. Song Y. Pan B. Fan B. (2023). Neuroprotective effects of longxue tongluo capsule on ischemic stroke rats revealed by LC-MS/MS-based metabolomics approach. Chin. Herb. Med. 15 (3), 430–438. 10.1016/j.chmed.2022.12.010 37538866 PMC10394346

[B79] Sylvain N. J. Salman M. M. Pushie M. J. Hou H. Meher V. Herlo R. (2021). The effects of trifluoperazine on brain edema, aquaporin-4 expression and metabolic markers during the acute phase of stroke using photothrombotic mouse model. Biochim. Biophys. Acta-Biomembr. 1863 (5), 183573. 10.1016/j.bbamem.2021.183573 33561476

[B80] Tan L. L. Huang R. S. Zhou G. Chen X. H. Liu Y. H. (2021). Clinical study on *Jianshen lishui* granule combined with furosemide in the treatment of secondary cerebral edema after intracerebral hemorrhage. China Med. Her. 18 (14), 72–75. 10.20047/j.issn1673-7210.2021.14.018

[B81] Techen N. Parveen I. Pan Z. Khan I. A. (2014). DNA barcoding of medicinal plant material for identification. Curr. Opin. Biotechnol. 25, 103–110. 10.1016/j.copbio.2013.09.010 24484887

[B82] Tsoi B. Chen X. Gao C. Wang S. Yuen S. C. Yang D. (2019a). Neuroprotective effects and hepatorenal toxicity of Angong Niuhuang Wan against ischemia-reperfusion brain injury in rats. Front. Pharmacol. 10, 593. 10.3389/fphar.2019.00593 31191319 PMC6548905

[B83] Tsoi B. Wang S. Gao C. Luo Y. Li W. Yang D. (2019b). Realgar and cinnabar are essential components contributing to neuroprotection of Angong Niuhuang Wan with no hepatorenal toxicity in transient ischemic brain injury. Toxicol. Appl. Pharmacol. 377, 114613. 10.1016/j.taap.2019.114613 31207256

[B84] Tyagi N. Qipshidze N. Munjal C. Vacek J. C. Metreveli N. Givvimani S. (2012). Tetrahydrocurcumin ameliorates homocysteinylated cytochrome-c mediated autophagy in hyperhomocysteinemia mice after cerebral ischemia. J. Mol. Neurosci. 47 (1), 128–138. 10.1007/s12031-011-9695-z 22212488 PMC3609416

[B85] Vakili A. Sharifat S. Akhavan M. M. Bandegi A. R. (2014). Effect of lavender oil (*Lavandula angustifolia*) on cerebral edema and its possible mechanisms in an experimental model of stroke. Brain Res. 1548, 56–62. 10.1016/j.brainres.2013.12.019 24384140

[B86] Wan Y. Holste K. G. Hua Y. Keep R. F. Xi G. (2023). Brain edema formation and therapy after intracerebral hemorrhage. Neurobiol. Dis. 176, 105948. 10.1016/j.nbd.2022.105948 36481437 PMC10013956

[B87] Wang J. (2018). Effect of Xueshuantong injection on neurological function, high-sensitivity c-reactive protein (hs-CRP) and matrix metalloproteinase-9 (MMP-9) in patients with intracerebral hemorrhage. Chin. J. Ration. Drug Explor. 15 (07), 24–27. 10.3969/j.issn.2096-3327.2018.07.008

[B88] Wang Y. F. Gu Y. T. Qin G. H. Zhong L. Meng Y. N. (2013). Curcumin ameliorates the permeability of the blood-brain barrier during hypoxia by upregulating heme oxygenase-1 expression in brain microvascular endothelial cells. J. Mol. Neurosci. 51 (2), 344–351. 10.1007/s12031-013-9989-4 23494637

[B89] Wang J. Li D. Hou J. Lei H. (2018). Protective effects of geniposide and ginsenoside rg1 combination treatment on rats following cerebral ischemia are mediated *via* microglial microRNA-155-5p inhibition. Mol. Med. Rep. 17 (2), 3186–3193. 10.3892/mmr.2017.8221 29257264

[B90] Wang X. F. Tian C. Zhang Y. Yuan M. C. Zhang H. L. Wang L. Q. (2019). Effect of naoxueshu oral liquid on the expression of zonula occludens-1 (ZO-1) and aquaporin 4 (AQP4) in brain tissue of rats with intracerebral hemorrhage model. China Med. Her. 16 (16), 8–12.

[B91] Wang Y. Xiao G. He S. Liu X. Zhu L. Yang X. (2020). Protection against acute cerebral ischemia/reperfusion injury by QiShenYiQi *via* neuroinflammatory network mobilization. Biomed. Pharmacother. 125, 109945. 10.1016/j.biopha.2020.109945 32028240

[B92] Wang J. Yin J. Zheng X. (2022a). Artemisinin upregulates neural cell adhesion molecule l1 to attenuate neurological deficits after intracerebral hemorrhage in mice. Brain Behav. 12 (5), e2558. 10.1002/brb3.2558 35349764 PMC9120716

[B93] Wang J. Zhang Y. Zhang M. Sun S. Zhong Y. Han L. (2022b). Feasibility of catalpol intranasal administration and its protective effect on acute cerebral ischemia in rats *via* anti-oxidative and anti-apoptotic mechanisms. Drug Des. Devel Ther. 16, 279–296. 10.2147/DDDT.S343928 PMC8801896 35115763

[B94] Wang P. Ren Q. Shi M. Liu Y. Bai H. Chang Y. Z. (2022c). Overexpression of mitochondrial ferritin enhances blood-brain barrier integrity following ischemic stroke in mice by maintaining iron homeostasis in endothelial cells. Antioxidants 11 (7), 1257. 10.3390/antiox11071257 35883748 PMC9312053

[B95] Wang X. Chen G. Wan B. Dong Z. Xue Y. Luo Q. (2022d). NRF1-mediated microglial activation triggers high-altitude cerebral edema. J. Mol. Cell Biol. 14 (5), mjac036. 10.1093/jmcb/mjac036 35704676 PMC9486928

[B96] Wang H. Chen Y. Wang L. Liu Q. Yang S. Wang C. (2023). Advancing herbal medicine: enhancing product quality and safety through robust quality control practices. Front. Pharmacol. 14, 1265178. 10.3389/fphar.2023.1265178 37818188 PMC10561302

[B97] Wang Z. Xue F. Zhang J. Wang Y. Hu E. Zheng Y. (2024a). The cornel iridoid glycoside attenuated brain edema of the cerebral ischemia/reperfusion rats by modulating the polarized aquaporin 4. Fitoterapia 177, 106098. 10.1016/j.fitote.2024.106098 38950636

[B98] Wang Z. Zhang X. Zhang G. Zheng Y. J. Zhao A. Jiang X. (2024b). Astrocyte modulation in cerebral ischemia-reperfusion injury: a promising therapeutic strategy. Exp. Neurol. 378, 114814. 10.1016/j.expneurol.2024.114814 38762094

[B99] Wang X. Shang Z. Zhao J. Hou H. Li Y. Song J. (2025). Efficacy and safety of early use of naoxueshu within 72 hours in the treatment of spontaneous intracerebral hemorrhage: a real-world retrospective cohort study. Int. J. Gen. Med. 18, 2057–2065. 10.2147/IJGM.S511802 40226803 PMC11994074

[B100] Wei D. Z. Liu H. Y. Wang Z. H. Chu J. Zhang M. Han H. Y. (2024). Clinical study on erigeron breviscapus injection combined with edaravone in the treatment of hypertensive intracerebral hemorrhage. Mod. Drugs Clin. 39 (12), 3099–3103. 10.7501/j.issn.1674-5515.2024.12.013

[B101] Widmann C. Gandin C. Petit-Paitel A. Lazdunski M. Heurteaux C. (2018). The traditional Chinese medicine MLC901 inhibits inflammation processes after focal cerebral ischemia. Sci. Rep. 8 (1), 18062. 10.1038/s41598-018-36138-0 30584250 PMC6305383

[B102] Xiang Y. Yao X. Wang X. Zhao H. Zou H. Wang L. (2019). Houshiheisan promotes angiogenesis *via* HIF-1α/VEGF and SDF-1/CXCR4 pathways: *in vivo* and *in vitro* . Biosci. Rep. 39 (10), BSR20191006. 10.1042/BSR20191006 31652450 PMC6822506

[B103] Xiao W. He Z. Luo W. Feng D. Wang Y. Tang T. (2021). BYHWD alleviates inflammatory response by NIK-mediated repression of the noncanonical NF-κB pathway during ICH recovery. Front. Pharmacol. 12, 632407. 10.3389/fphar.2021.632407 34025405 PMC8138445

[B104] Xiao H. Liu S. Fang B. Zhang W. Wang M. Ye J. (2024). Panax notoginseng saponins promotes angiogenesis after cerebral ischemia-reperfusion injury. J. Ginseng Res. 48 (6), 592–602. 10.1016/j.jgr.2024.08.004 39583172 PMC11584196

[B105] Xie Y. Zhang W. Wang H. Hu H. Zhang S. Wang S. (2025). Application of physiologically based pharmacokinetic modeling in the research of anti-HIV drugs. Curr. Drug Metab. 26, 472–488. 10.2174/0113892002392579250902053006 40968422

[B106] Xue H. Feng Z. Jin C. Zhang Y. Ai Y. Wang J. (2025). Soy isoflavones protects against stroke by inhibiting keap1/NQO1/nrf2/HO-1 signaling pathway: network pharmacology analysis combined with the experimental validation. Pharmaceuticals 18 (4), 548. 10.3390/ph18040548 40283984 PMC12030689

[B107] Yang B. Sun Y. Lv C. Zhang W. Chen Y. (2020). Procyanidins exhibits neuroprotective activities against cerebral ischemia reperfusion injury by inhibiting TLR4-NLRP3 inflammasome signal pathway. Psychopharmacologia 237 (11), 3283–3293. 10.1007/s00213-020-05610-z 32729095

[B108] Yang Y. Gao H. Liu W. Liu X. Jiang X. Li X. (2021). Arctium lappa l. roots ameliorates cerebral ischemia through inhibiting neuronal apoptosis and suppressing AMPK/mTOR-mediated autophagy. Phytomedicine 85, 153526. 10.1016/j.phymed.2021.153526 33691269

[B109] Yang Y. Li L. Yu L. Xia Y. Fang Z. Wang S. (2024). Naringenin protected against blood brain barrier breakdown after ischemic stroke through GSK-3β/β-Catenin pathway. Neurochem. Res. 50 (1), 17. 10.1007/s11064-024-04259-w 39556287

[B110] Yao Y. H. (2012). Clinical study on early application of shuxuetong injection in the treatment of intracerebral hemorrhage. Chin. J. Clin. Res. 25 (01), 38–39.

[B111] Yu Z. S. Xu D. Y. Yang Y. L. Pang Q. Q. (2011). Systematic review of total saponins of Panax notoginseng injection in the treatment of acute intracerebral hemorrhage. J. Youjiang Med. Univ. Natl. 33 (01), 19–23. 10.3969/j.issn.1001-5817.2011.01.008

[B112] Yu W. Y. Yu J. B. Luo G. (2018). Clinical study on longhu xingnao granule in preventing cerebral edema formation after acute intracerebral hemorrhage. Guid. J. Traditional Chin. Med. Pharm. 24 (06), 63–65. 10.13862/j.cnki.cn43-1446/r.2018.06.021

[B113] Yu J. Lü Y. Li Z. M. Yu X. (2023). Clinical study on sanyu tongluo decoction in the treatment of intracerebral hemorrhage. Chin. J. Integr. Med. Cardio-Cerebrovascular Dis. 21 (21), 4006–4010. 10.12102/j.issn.1672-1349.2023.21.028

[B114] Yu J. Zhou H. Guo J. Chen T. Shao C. Pan Z. (2025). Zhongfeng Xingnao prescription alleviates injury of intracerebral hemorrhage *via* regulating the CaMKII/NF-κB p65/NLRP3/GSDMD signaling axis. J. Tradit. Complement. Med. 15 (1), 84–92. 10.1016/j.jtcme.2024.03.005 39807270 PMC11725133

[B115] Zeng J. (2015). Clinical observation on salvia miltiorrhiza injection combined with nimodipine in the treatment of hypertensive intracerebral hemorrhage. Forum Prim. Med. 19 (04), 490–491.

[B116] Zeng X. Zhang S. Zhang L. Zhang K. Zheng X. (2006). A study of the neuroprotective effect of the phenolic glucoside gastrodin during cerebral ischemia *in vivo* and *in vitro* . Planta Med. 72 (15), 1359–1365. 10.1055/s-2006-951709 17089323

[B117] Zeng M. Zhou H. He Y. Wang Z. Shao C. Yin J. (2021). Danhong injection alleviates cerebral ischemia/reperfusion injury by improving intracellular energy metabolism coupling in the ischemic penumbra. Biomed. Pharmacother. 140, 111771. 10.1016/j.biopha.2021.111771 34058441

[B118] Zhang S. X. Chen J. Y. (2016). Effect of angong niuhuang wan on serum nitric oxide (NO) and asymmetric dimethylarginine (ADMA) in comatose patients with acute cerebral infarction and its clinical efficacy observation. Mod. J. Integr. Traditional Chin. West. Med. 25 (17), 1873–1875. 10.3969/j.issn.1008-8849.2016.17.017

[B119] Zhang X. Chen L. Dang X. Liu J. Ito Y. Sun W. (2014). Neuroprotective effects of total steroid saponins on cerebral ischemia injuries in an animal model of focal ischemia/reperfusion. Planta Med. 80 (8-9), 637–644. 10.1055/s-0034-1368584 24963614 PMC4083247

[B120] Zhang X. Liu X. Hu G. Zhang G. Zhao G. Shi M. (2020). Ginsenoside rd attenuates blood-brain barrier damage by suppressing proteasome-mediated signaling after transient forebrain ischemia. Neuroreport 31 (6), 466–472. 10.1097/WNR.0000000000001426 32168101

[B121] Zhang B. Zeng Z. Wu H. (2021). A network pharmacology-based analysis of the protective mechanism of miao medicine xuemaitong capsule against secondary brain damage in the ischemic area surrounding intracerebral hemorrhage. J. Pharmacol. Exp. Ther. 377 (1), 86–99. 10.1124/jpet.120.000083 33310816

[B122] Zhang C. Shi Z. Xu Q. He J. Chen L. Lu Z. (2023). Astragaloside IV alleviates stroke-triggered early brain injury by modulating neuroinflammation and ferroptosis *via* the nrf2/HO-1 signaling pathway. Acta Cir. Bras. 38, e380723. 10.1590/acb380723 36995819 PMC10041803

[B123] Zhao Y. C. Wang T. Gao Y. P. (2010). Clinical observation of huoxue tongli decoction in the treatment of acute intracerebral hemorrhage. China J. Traditional Chin. Med. Pharm. 25 (10), 1697–1699.

[B124] Zhao B. Shi Q. J. Zhang Z. Z. Wang S. Y. Wang X. Wang H. (2018). Protective effects of paeonol on subacute/chronic brain injury during cerebral ischemia in rats. Exp. Ther. Med. 15 (4), 3836–3846. 10.3892/etm.2018.5893 29563983 PMC5858057

[B125] Zhao G. F. Liu D. L. Xu Y. J. Wang J. Y. Wang J. (2023). Clinical effect of xiaoxuming decoction in the treatment of cerebral edema after large-area cerebral infarction. Chin. Archives Traditional Chin. Med. 41 (01), 179–182. 10.13193/j.issn.1673-7717.2023.01.040

[B126] Zhao Y. Cui W. Xie T. Zhao K. Li Y. Wan Y. (2024). Efficacy and safety of Chinese herbal medicine in patients with acute intracerebral hemorrhage: protocol for a randomized placebo-controlled double-blinded multicenter trial. Cerebrovasc. Dis. 53 (4), 501–508. 10.1159/000534761 39250890

[B127] Zheng Y. Q. Liu J. X. Wang J. N. Xu L. (2007). Effects of crocin on reperfusion-induced oxidative/nitrative injury to cerebral microvessels after global cerebral ischemia. Brain Res. 1138, 86–94. 10.1016/j.brainres.2006.12.064 17274961

[B128] Zheng L. Meng L. Liang H. Yang J. (2023). Sanhua decoction: current understanding of a traditional herbal recipe for stroke. Front. Neurosci. 17, 1149833. 10.3389/fnins.2023.1149833 37123364 PMC10133510

[B129] Zheng F. Guo X. Yan Q. Zhou Y. Hu E. Zhu H. (2025). Hydroxysafflor yellow a attenuates the blood-brain barrier dysfunction and neuroinflammation through anti-inflammatory microglial polarization after intracerebral hemorrhage. Neuropharmacology 278, 110576. 10.1016/j.neuropharm.2025.110576 40571121

[B130] Zhong X. Y. Yang W. Q. Ye R. H. (2019). Clinical study on liangxue sanyu decoction combined with nimodipine in the treatment of ischemic brain injury after intracerebral hemorrhage. China Med. Pharm. 9 (05), 57–59.

[B131] Zhu H. Wang Z. Xing Y. Gao Y. Ma T. Lou L. (2012). Baicalin reduces the permeability of the blood-brain barrier during hypoxia *in vitro* by increasing the expression of tight junction proteins in brain microvascular endothelial cells. J. Ethnopharmacol. 141 (2), 714–720. 10.1016/j.jep.2011.08.063 21920425

