# Supplementary material for: Selected traditional Chinese medicine interventions for post-stroke cerebral edema: a review integrating clinical evidence and mechanistic insights
Source: Front Pharmacol. 2025 Dec 10;16:1709821. doi: 10.3389/fphar.2025.1709821 (PMC12727903; doi:10.3389/fphar.2025.1709821)
Supplement: Supplementary file 1 [file Supplementaryfile1.docx]

**Web of Science**

(TS=(ischemia) OR TS=(ischemic stroke) OR TS=(Cerebral hemorrhage) OR TS=(intracerebral hemorrhage) OR TS=(ICH) OR TS=(stroke)) AND (TS=(monomer) OR TS=(Chinese medicine) OR TS=(TCM) OR TS=(Active ingredient) OR TS=(natural products) OR TS=(Biomolecules) OR TS=(herbal) OR TS=(active component) OR TS=(natural product) OR TS=(phytochemical)) AND TS=(edema)

**PUBMED**

((ischemia[Title/Abstract]) OR (ischemic stroke [Title/Abstract]) OR (Cerebral hemorrhage [Title/Abstract]) OR (intracerebral hemorrhage[Title/Abstract]) OR (ICH[Title/Abstract]) OR(stroke[Title/Abstract])) AND ((monomer[Title/Abstract]) OR (Chinese medicine [Title/Abstract]) OR (TCM[Title/Abstract]) OR (Active ingredient [Title/Abstract]) OR (natural products [Title/Abstract]) OR (Biomolecules [Title/Abstract]) OR (herbal [Title/Abstract]) OR (active component [Title/Abstract]) OR (natural product [Title/Abstract]) OR (phytochemical [Title/Abstract])) AND (edema [Title/Abstract])

**China National Knowledge Infrastructure (CNKI)**

(SU='卒中' OR SU='脑缺血' OR SU='脑出血' OR SU='中风' OR SU='缺血性卒中' OR SU='出血性卒中') AND (SU='脑水肿' OR SU='水肿') AND (SU='中药' OR SU='中医药' OR SU='中草药' OR SU='草药' OR SU='中成药' OR SU='方剂' OR SU='有效成分' OR SU='提取物' OR SU='单体')

(SU='stroke' OR SU='cerebral ischemia' OR SU='intracerebral hemorrhage' OR SU='apoplexy' OR SU='ischemic stroke' OR SU='hemorrhagic stroke') AND (SU='cerebral edema' OR SU='edema') AND (SU='traditional Chinese medicine' OR SU='Chinese herbal medicine' OR SU='herbal medicine' OR SU='Chinese patent medicine' OR SU='formula' OR SU='active ingredient' OR SU='extract' OR SU='monomer')

**VIP**

(M=卒中 OR M=脑缺血 OR M=脑出血 OR M=中风 OR M=缺血性卒中 OR M=出血性卒中) AND (M=脑水肿 OR M=水肿) AND (M=中药 OR M=中医药 OR M=中草药 OR M=草药 OR M=中成药 OR M=方剂 OR M=有效成分 OR M=提取物 OR M=单体)

(M='stroke' OR M='cerebral ischemia' OR M='intracerebral hemorrhage' OR M='apoplexy' OR M='ischemic stroke' OR M='hemorrhagic stroke') AND (M='cerebral edema' OR M='edema') AND (M='traditional Chinese medicine' OR M='Chinese herbal medicine' OR M='herbal medicine' OR M='Chinese patent medicine' OR M='formula' OR M='active ingredient' OR M='extract' OR M='monomer')

**Wanfang**

主题:(卒中 OR 脑缺血 OR 脑出血 OR 中风 OR 缺血性卒中 OR 出血性卒中) AND 主题:(脑水肿 OR 水肿) AND 主题:(中药 OR 中医药 OR 中草药 OR 草药 OR 中成药 OR 方剂 OR 有效成分 OR 提取物 OR 单体)

Subject:(stroke OR cerebral ischemia OR intracerebral hemorrhage OR apoplexy OR ischemic stroke OR hemorrhagic stroke) AND Subject:(cerebral edema OR edema) AND Subject:(traditional Chinese medicine OR Chinese herbal medicine OR herbal medicine OR Chinese patent medicine OR formula OR active ingredient OR extract OR monomer)

**SinoMed**

(("卒中"[常用字段] OR "脑缺血"[常用字段] OR "脑出血"[常用字段] OR "中风"[常用字段] OR "缺血性卒中"[常用字段] OR "出血性卒中"[常用字段])) AND (("脑水肿"[常用字段] OR "水肿"[常用字段])) AND (("中药"[常用字段] OR "中医药"[常用字段] OR "中草药"[常用字段] OR "草药"[常用字段] OR "中成药"[常用字段] OR "方剂"[常用字段] OR "有效成分"[常用字段] OR "提取物"[常用字段] OR "单体"[常用字段]))

(("stroke"[common field] OR "cerebral ischemia"[common field] OR "intracerebral hemorrhage"[common field] OR "apoplexy"[common field] OR "ischemic stroke"[common field] OR "hemorrhagic stroke"[common field])) AND (("cerebral edema"[common field] OR "edema"[common field])) AND (("traditional Chinese medicine"[common field] OR "Chinese herbal medicine"[common field] OR "herbal medicine"[common field] OR "Chinese patent medicine"[common field] OR "formula"[common field] OR "active ingredient"[common field] OR "extract"[common field] OR "monomer"[common field]))

**Identification of studies via databases and registers**

A total of 3,267 records were identified through database searching

CNKI (n=548), VIP (n=115), Wanfang Data (n=1,240),

Sinomed (n=934), PubMed (n=163), Web of Science (n=267).

Search Date: Through 23 July 2025

**Identification**

**Exclusion Criteria for the Clinical Study**
(a) Case reports, review articles, commentaries, and animal studies.
(b) Patients with strokes other than cerebral ischemia or intracerebral hemorrhage.
(c) Other complementary and alternative therapies, including acupuncture, moxibustion, cupping, tuina massage, qigong, tai chi, yoga, and music therapy.
(d) Studies that did not report clinical outcomes.对于临床研究，排除标准如下:(a)病例报告、综述、评论和动物研究; (b)非脑缺血或脑出血患者;(c)其他补充和替代疗法，包括针灸、拔罐、推拿、和音乐疗法 (d)未报告临床结局。

**Exclusion Criteria for Preclinical Studies**
(a) Case reports, review articles, commentaries, and clinical studies.
(b) Studies utilizing cell or animal models unrelated to stroke.
(c) Other complementary and alternative therapies, including acupuncture, cupping, tuina massage, and music therapy.
(d) Studies that did not report experiment outcomes related to cerebral edema.于临床研究，排除标准如下:(a)病例报告、综述、评论和动物研究; (b)非脑缺血或脑出血患者;(c)其他补充和替代疗法，包括针灸、拔罐、推拿、和音乐疗法 (d)未报告临床结局。

**Screening**

Thirty-six articles were included in the final selection.

**Included**

Seventeen articles were included in the final selection.

1. **Flow chart of literature screening.**
