# Supplementary material for: Selected traditional Chinese medicine interventions for post-stroke cerebral edema: a review integrating clinical evidence and mechanistic insights
Source: Front Pharmacol. 2025 Dec 10;16:1709821. doi: 10.3389/fphar.2025.1709821 (PMC12727903; doi:10.3389/fphar.2025.1709821)
Supplement: Supplementary file 2 [file Supplementaryfile2.docx]

| Evidence domain | Main issues identified | Overall quality | Certainty of evidence | Remarks |
| --- | --- | --- | --- | --- |
| Study design | Most trials are small-sample, single-center studies; few are randomized with adequate controls | Low–moderate | Limited | Lacks large-scale, multicenter RCTs |
| Risk of bias | Incomplete blinding, unclear allocation concealment, and inconsistent AE reporting | Moderate–high | Limited | Bias may overestimate efficacy |
| Consistency | Generally consistent direction of benefit across studies | Moderate | Moderate | Magnitude of effect varies |
| Directness | Most studies directly assess cerebral edema or neurological outcomes | Moderate | Moderate | Some indirect endpoints (e.g., MRI markers) |
| Precision | Wide confidence intervals and small sample sizes | Low | Limited | Requires replication |
| Publication bias | Likely present (few negative studies published) | High | Limited | Acknowledged limitation |

**Note:** Evidence quality was summarized qualitatively according to the conceptual domains of the GRADE framework.

Given the narrative nature of this review and high study heterogeneity, a formal GRADE scoring was not applicable.
